# Review on Cellular Automata for Microstructure Simulation of Metallic Materials

**DOI:** 10.3390/ma17061370

**Published:** 2024-03-16

**Authors:** Ying Zhi, Yao Jiang, Diwen Ke, Xianlei Hu, Xianghua Liu

**Affiliations:** 1 State Key Laboratory of Rolling and Automation, Northeastern University, Shenyang 110819, China; 2 School of Material Science and Engineering, Northeastern University, Shenyang 110819, China

**Keywords:** cellular automata, solidification, recrystallization, phase transformation, carbide precipitation, additive manufacturing, welding, corrosion

## Abstract

The cellular automata (CA) method has played an important role in the research and development of metallic materials. CA can interpret the microstructure changes of materials and obtain more abundant, accurate and intuitive information of microstructure evolution than conventional methods. CA can visually represent the process of grain formation, growth, development and change to us in a graphical way, which can assist us in analysis, thinking and solving problems. In the last five years, the application of CA in materials research has been rapidly developed, and CA has begun to occupy an increasingly important position in the simulation research of metallic materials. After introducing the advantages and limitations of CA compared to other widely used simulation methods, the purpose of this paper is to review the recent application progress on the microstructure simulation of metallic materials using CA, such as solidification, recrystallization, phase transformation and carbide precipitation occurring during forming and heat treatment. Specifically, recent research advances on microstructure simulation by CA in the fields of additive manufacturing, welding, asymmetrical rolling, corrosion prevention, etc., are also elaborated in this paper. Furthermore, this paper points out the future work direction of CA simulation in the research of metallic materials, especially in the simulation of the crystal structure, the prediction of mechanical properties, CA simulation software and rule systems, etc. These are expected to attract wide attention of researchers in the field of metallic materials and promote the development of CA in materials research.

## 1. Introduction

With the rapid development of computer technology, numerical simulation plays a vital role in the research and development of metallic materials. Traditional methods of material research rely on many repeated experiments and existing experience and principles, which are increasingly becoming bottlenecks in the development of new materials and technological progress. However, with the help of existing efficient and accurate simulation technology, combined with many experimental data analyses, the development cycle of the material can be shortened and the costs of the research and development can be reduced [1,2]. In this paper, the cellular automata (CA) method, like the phase field method and the Monte Carlo method, is an effective way to simulate and study the microstructure evolution of metallic materials, including steel materials such as stainless steel and high strength steel and lightweight metallic materials such as magnesium alloy and aluminum alloy. CA have played an important role in the research and development of metallic materials [3,4].

Cellular automata are a dynamic system with discrete time and space that can simulate the spatiotemporal evolution of complex systems. Since the 21st century, CA have become the frontier of academic research; relevant scholars have conducted in-depth research on the theory and application of CA. In the field of metallic materials research, scholars have also applied CA to the study of the microstructural simulation in the process of metal formation and heat treatment [5,6]. CA can interpret the microstructure changes of metallic materials and obtain more abundant, accurate and intuitive information of microstructure evolution than conventional methods. It has been used in the simulation of metal microstructure evolution such as metal solidification, recrystallization and phase transformation. New progress in CA applications in microstructure simulation in metallic materials research has brought about a series of important changes in this field [7,8]. In the past, the research on grain and its deformation was mostly based on hypothesis, simplification, observation and deduction, and mathematical formulas were established as descriptive means. However, CA can visually present the process of grain formation, growth, development and change to us in a graphical way, which can assist us in analysis, thinking and solving problems.

Although the traditional theory can explain some phenomena about grain size and grain deformation, it is very difficult to simulate grain shape. CA can not only give average grain size and size distribution, but also give more abundant information about grain shape and orientation in graphical way. CA allow us to see grain boundaries directly and to see the formation, expansion, evolution, fusion and disappearance of grain boundaries, which is undoubtedly a huge impetus to our research on grain boundaries. CA can provide us with dynamic images of the microstructure evolution of metallic materials. How does the solidification microstructure of metals come into being? How does the grain change during rolling? How does recrystallization and phase transformation proceed? These are important issues related to the evolution process. In the past, only a few clues could be captured to imagine, or a clever animation could be used to illustrate them. Nowadays, these processes can be clearly displayed on the computer screen by CA, just like watching a revelation movie.

CA are such a powerful, interesting and promising tool, and scholars of metallic materials have no reason to ignore it. In this paper, we introduce the advantages and limitations of CA compared to other widely used simulation programs. Subsequently, this paper is to conduct a focused review on the recent progress of CA simulation of microstructure evolution of metallic materials in the field of solidification, recrystallization, phase transformation and carbide precipitation occurring during forming and heat treatment in the last five years. Specifically, recent research advances on the microstructure simulation by CA in the fields of additive manufacturing, welding, asymmetrical rolling, corrosion prevention, etc., are also elucidated in this paper. At the same time, the future work direction of CA simulation to the microstructure evolution of metallic materials is also presented. Since CA can provide a theoretical basis for the development of metallic materials and production processes, this paper aims to attract more scholars to pay attention to cellular automata and apply it to the development and research of metallic materials. The CA method, as a new method of scientific research in addition to experiments, has greatly promoted the development and practical application of new materials. It not only provides a new perspective for the researchers of materials science in the research method, but also significantly promotes the development of materials science.

## 2. CA Characteristics and Modeling Steps for Microstructure Simulation

### 2.1. CA Characteristics and Comparison with Other Microstructure Simulation Methods

Cellular automata, first proposed by the mathematician Neumann [9], are a dynamic system in which space, time and system states are all considered discrete. Space is divided into many units by a certain format of grid, each of which is called a cell. In space, the evolutionary state of cells evolves according to a regional evolution rule. Moreover, within discrete time steps, all cells are updated synchronously, thereby changing the state of the entire spatial cell [10]. Furthermore, it is worth noting that CA are not determined by any function or physical equation; rather, it exists as a general method framework. Consequently, any model that satisfies these rules can be called a cellular automaton model [9]. Moreover, in the field of microstructure simulation, the CA method describes the evolution process of microstructures, primarily through transformation rules. Additionally, CA is suitable for reproducing microstructure evolution [11] as well as for constructing dynamic models [12]. Furthermore, besides the CA method, other major microstructure simulation methods include the Monte Carlo (MC) method and the phase field (PF) method. Each of these three simulation methods has its own characteristics and applied range. The MC method, which falls under the category of stochastic statistical mathematics, is particularly well-suited for simulating two-dimensional or three-dimensional grain growth [13], and it also models grain topology and growth kinetics effectively [14]. The fundamental principle of the PF method is to use a continuous field to describe interfaces, making it especially appropriate for microstructure simulation such as metal solidification and dendritic growth [15].

**Figure 1:**
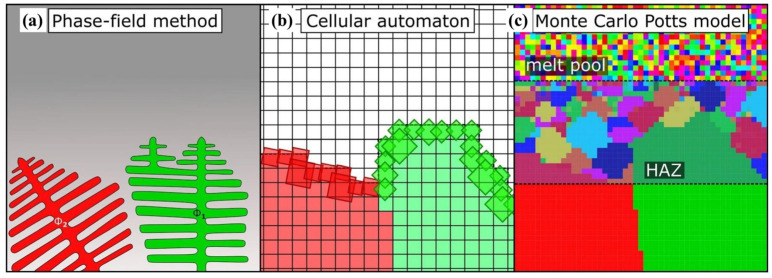
Schematic of the different modeling approaches: (**a**) phase field method; (**b**) cellular automaton; (**c**) Monte Carlo Potts model [18].

Each of these three microstructure simulation methods has its own advantages and limitations. Although the MC method has advantages in computational efficiency, it has limitations, such as a lack of a physical background, limitations in simulating phase transformation dynamics and the ambiguity of the relationship between time steps and actuality correspondence [16]. In contrast, the CA method is more computationally efficient compared to the PF method. However, the CA method’s accuracy is greatly influenced by the shape and size of cells, with a notable increase in error, particularly when cell sizes exceed 1 μm. The PF method is advantageous for its higher simulation accuracy, but its computational efficiency is low due to the large number of equations to be solved, especially when dealing with large-scale microstructure evolution [16]. Additionally, the spatial scales associated with these three microstructure simulation methods also differ significantly. The spatial scale for the CA method ranges from 10^−10^ to 10^−6^ m [17], for the MC method it ranges from 10^−10^ to 10^−6^ m [17], and for the PF method it ranges from 10^−9^ to 10^−5^ m [17]. Clearly, the CA method has the widest span in spatial scale. To offer a more vivid depiction of the distinctions between the three simulation methods, Figure 1 shows the fundamental concepts of various microstructure modeling methods, including PF, CA and MC models [18]. Among them, Figure 1a shows that the core principle of the phase field method is a smoothly varying function, called phase field, describing the interface between two flowing phases. Figure 1b shows that in the CA method, the physical system of interest is divided into cells, where each cell is allowed to interact iteratively with its neighbors according to some specific rules. Figure 1c indicates grain structure evolution during the solidification process established by the MC method.

Differing from microscale simulation methods, mesoscale simulation methods include the crystal plasticity finite element method (CPFEM), which is an effective tool for studying the deformation processes and mechanical behaviors of metallic materials at both macro and micro scales. It is primarily applied in the simulation or prediction of mechanical properties of metallic materials [19,20,21]. The spatial scale for the CPFEM ranges from 10^−6^ to 10^0^ m [17]. In comparison with the CA method, the CPFEM has more limitations in simulating microstructure evolution. It focuses more on crystal plasticity mechanics and the changes in crystal orientation during the simulation of deformation processes. Unlike the CPFEM, the general finite element method (FEM) is limited to solving macroscopic problems, with a corresponding spatial scale ranging from 10^−5^ to 10^0^ m [17]. Compared with the CA method, general FEM emphasizes simulating macroscopic stress–strain fields, temperature fields, etc., during material deformation and heat treatment processes, but it struggles to simulate changes in microstructure. Therefore, the FEM is often combined with the CA method [22,23], the MC method [24,25] and the PF method [26] to create new models that can simulate microstructure.

**Figure 2:**
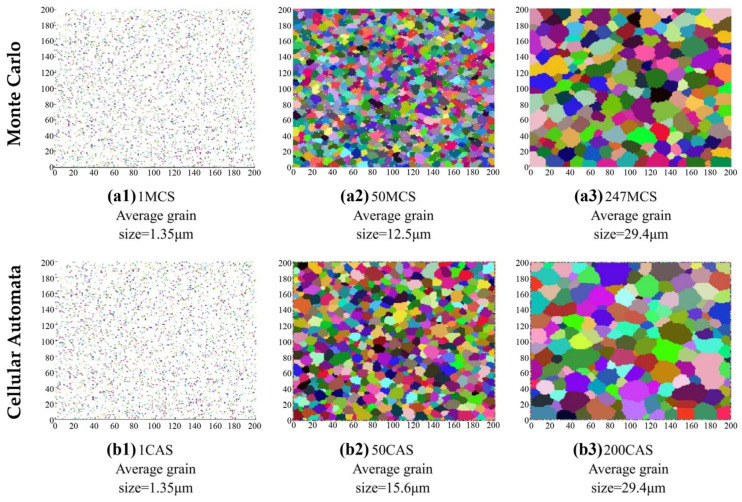
(**a1**–**a3**): Nucleation and grain growth of 6061 in LAM by the Monte Carlo method: (**a1**) 1MCS; (**a2**) 50MCS; (**a3**) 247MCS. (**b1**–**b3**): nucleation and grain growth of 6061 in LAM by the CA method: (**b1**) 1CAS; (**b2**) 50CAS; (**b3**) 200CAS. Different colors represent different grains [28].

The Monte Carlo model is established following probabilistic strategic on selections of grain coarsening orientations, which makes it highly efficient for the problems related to multi-length and time scales [27]. When comparing the CA method with the MC method, CA has unparalleled advantages, including simpler algorithms and higher computational efficiency. The research [20] has indicated that the CA method has a higher computational efficiency compared to the MC method. In the field of additive manufacturing, scholars have used both the MC and CA methods to study the microstructure evolution of nucleation and grain growth in 6061 aluminum alloy during laser additive manufacturing (LAM). When the MC method has been used to simulate grain nucleation and growth, one lattice point was randomly selected, and its state and orientation may be changed depending on its neighborhoods. Conversely, the grain growth was controlled by the selected strategy, i.e., the neighborhood types when using the CA method. According to the simulation results by these two different simulation methods, it was found that when Cellular Automaton Step (CAS) was 200, the predicted average grain size could reach the final state shown by 247 Monte Carlo Step (MCS). This suggested that fewer CAS were needed to achieve the same grain size and morphology as the MC method, meaning that CA has higher computational efficiency, as shown in Figure 2 [28]. In terms of static recrystallization kinetics, scholars have established two models using both the MC and CA methods, respectively. Both the MC and CA models were capable of correctly reproducing the static recrystallization kinetics. However, in the MC model, an important parameter—the H/J ratio, where H represented the energy stored in the lattice site and J denoted the grain boundary energy—described the level of deformation and recrystallization temperature. The high values of the H/J ratio resulted in an unphysical shape of the recrystallization front. In contrast, the CA model did not suffer from that limitation. Additionally, CA could also accurately track the recrystallization front, and the simulation steps were based on analytical equations with real-time constraints [29]. In summary, the scholar believes that the CA method is more attractive in practice, while the practical value of the MC model is limited [29].

**Figure 3:**
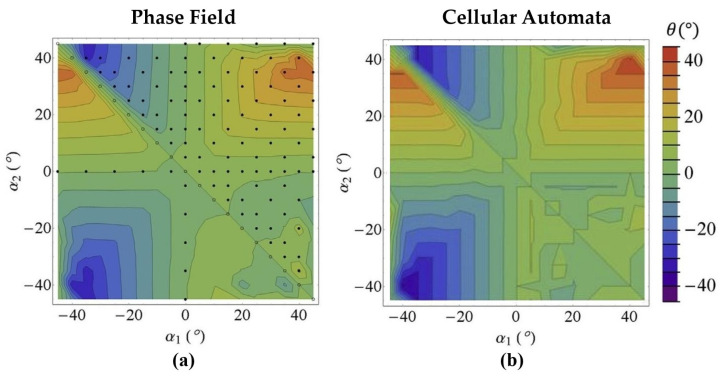
Comparison of predicted grain boundary orientations by different simulation methods: (**a**) phase field method [35,36]; (**b**) cellular automata method [34]. Black dots symbols in (**a**) represent the 107 simulations couples (α1, α2). Different colors represent different grain boundary orientations.

The phase field method is a simulation technique grounded in thermodynamics and dynamics. It evolves phase field variables such as order parameters, temperature, concentration, etc., by minimizing the total energy of the system [30]. This method is notable for its high precision and resolution in representing physical models, allowing it to accurately capture sub-grain characteristics within solidification microstructure [31]. A significant advantage of the PF method is its capacity to simulate the real time evolution of microstructures towards thermodynamic equilibrium, in comparison to MC and CA, where the steps can only afterwards be related to time [28]. Despite its advantages in simulation precision, the PF method has a significant drawback: higher computational costs. This limitation has primarily confined the use of the PF method to research focusing on the growth dynamics of very small-scale multicomponent alloys [32]. In comparison, the CA method results in lower computational costs due to coarser discretization and relatively local and simple computations [33]. The advantage of lower computational costs associated with CA have also been confirmed in subsequent literature. In the solidification microstructure simulation, the grain boundary (GB) orientation during directional solidification of a bi-crystal of a succinonitrile—0.4 wt% acetone alloy has been predicted using the CA methods [34]. Furthermore, the CA simulation results were compared with the PF simulation results under identical conditions. There were some differences between these two simulation methods regarding the GB orientation. Specifically, the PF method [35,36] considered the deviation of the growth direction from the <10> crystallographic directions for low Peclet numbers when simulating the GB orientation, while the CA method neglected the influence of the Peclet number on the <10> crystallographic directions when simulating the GB orientation. The comparison of the simulation results shown in Figure 3 indicated that the GB orientations predicted by both the CA and PF methods were very consistent. However, when comparing required computational resources between the CA and PF methods, the advantage of the CA method was obvious, and CA could reach higher length and time scales. This advantage was evident when comparing the computational resources needed by each method. The PF simulations for a given selection map (Figure 3a) [35,36] took approximately 255 days, while the same selection map took only 6 h for the CA simulation on a single desktop computer processor (Figure 3b) [34]. To this day, the CA method remains the preferred approach for predicting grain structure in industrial casting processes due to these advantages [37].

### 2.2. CA Modeling Approach and Steps for Microstructure Simulation

Cellular automata are also translated into lattice automata or molecular automata, which are discrete dynamical systems in time and space. Each cell scattered in the regular grid takes finite discrete states and follows the same rules of action, updating synchronously according to the determined local rules. Many cells complete the evolution of the dynamic system through simple interactions. The basic components of CA consist of four parts: cell, cell space, neighbor and rule [38,39]. In general, CA can be considered to consist of cellular space and evolution rule defined in the space.

**Figure 4:**
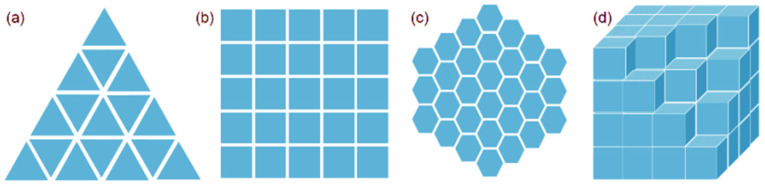
Cell types of 2-D and 3-D cellular automaton. (**a**) Triangular cell; (**b**) quadrilateral cell; (**c**) hexagonal cell; (**d**) hexahedral cell [39].

**Figure 5:**
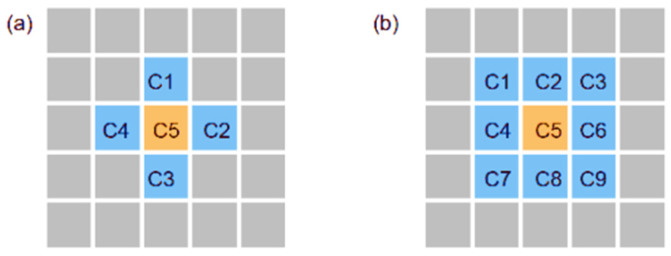
Neighbor types of 2-D cellular automaton. (**a**) Von Neumann neighbor type; (**b**) Moore neighbor type [39].

The steps of cellular automata to simulate the microstructure evolution of the metallic materials can be summarized as follows:

(1) The first step is to establish the physical metallurgy mathematical model of metal solidification, recrystallization or phase transformation, etc.
(2) The second step is to build the CA model of metal microstructure evolution. In this step, it is important to select the cell type and the neighbor type; Figure 4 and Figure 5 are diagrams of common cell types and neighbor types, respectively [39]. In addition, determining the metastable transition rules is crucial for the success of the microstructure simulation. In the actual research process, the evolution rules of cells are set according to the research problems and are changeable. The evolution rules of CA determine the state of every cell at every moment. Evolution rules can be deterministic or random. On the basis of the actual situation, it is extremely creative to determine the appropriate evolution rules, which is based on a full understanding of the real physical mechanism and the macro process of the system.
(3) The last step is to write the simulation program using MATLAB (https://www.mathworks.com), PYTHON (https://www.python.org), FORTRAN (https://www.intel.cn), or other program languages to realize the simulation of microstructure evolution and visual results.

## 3. Application Progress of CA in Microstructure Simulation

Cellular automata have been widely used in the microstructure simulation of metallic materials. CA have played an important role in the application of microstructure simulation of metal solidification, recrystallization and phase transformation. The physical metallurgy mathematical models of metal solidification, recrystallization, phase transformation or carbide precipitation are established to simulate microstructure evolution, which can obtain important information such as grain size, microstructure shape and distribution, volume fraction, dislocation density, etc. In this paper, CA have been an effective method to explore the law and mechanism for microstructure evolution of various metallic materials, including steel materials such as stainless steel and high-strength steel, and lightweight metallic materials such as magnesium alloy and aluminum alloy. Recently, it is noteworthy that CA have begun to occupy an increasingly important position in the simulation research of metallic materials, such as additive manufacturing, welding, asymmetrical rolling, corrosion prevention, etc.

### 3.1. Application of CA in Solidification Simulation

Solidification of metal refers to a phase transformation process from liquid to solid, which occurs once or several times during the preparation process of most metallic materials. Solidification microstructure not only affects the subsequent hot working process of the material, but also directly influences the final mechanical properties of the material. Particularly, with the rapid development of simulation computing technology, numerical simulation has become the third scientific research method, developing in parallel with experimental and theoretical research. As one of the essential simulation and modeling tools, CA was first applied to simulate microstructure evolution of solidification processes in materials science. In 1986, Packard [40] established the first two-dimensional (2-D) CA model of dendrite growth, which made people see that the CA method, as a new research method, could provide a reproduction of the continuous change process of metal solidification that could not be achieved in experiments. On the basis of this research, a growing number of scholars have joined in the study of using the CA method to simulate the metal solidification microstructure and have carried out much research in the field. Since the evolution law of solidification microstructure can be obtained through CA simulation, which can then provide theoretical guidance, the CA method has evolved quite rapidly in the field of solidification. Recently, the CA models developed can be used for solidification simulations, which are common in casting, welding and additive manufacturing.

#### 3.1.1. CA Simulation in Casting

In the last five years, CA simulation has yielded many research results in casting, mainly focusing on dendrite growth, porosity, eutectic transformation during solidification, and the influence of various process parameters on microstructure evolution and porosity defects.

**Figure 6:**
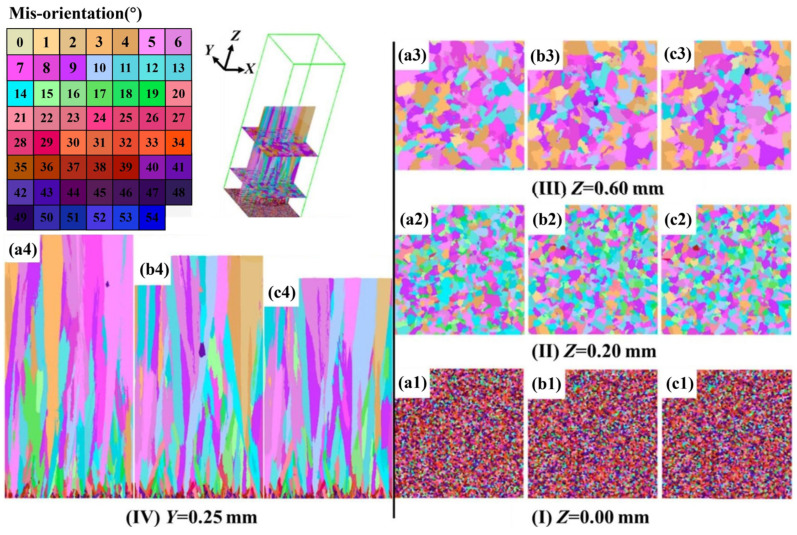
Solidification structure of Y and Z sections with different flow intensities: (**a1**–**c1**) Z = 0.00 mm section; (**a2**–**c2**) Z = 0.20 mm section; (**a3**–**c3**) Z = 0.60 mm section; (**a4**–**c4**) Y = 0.25 mm section. (**a1**–**a4**) Non-flow; (**b1**–**b4**) free convection; (**c1**–**c4**) forced convection. Different colors represent different mis-orientations [41].

The two-roll strip casting process is an advanced process that combines the two steps of casting and rolling into a one-step process, which involves the phenomenon of solidification of the steel as it dissipates heat through its interface with the casting rolls. Since casting parameters are difficult to realize accurate control only by experience, this has produced a great challenge of how to ensure the quality of the strip during the solidification process. Bai et al. [41] yielded insights into the influence of each casting parameter on the solidification microstructure of two-roll strip casting from the perspective of numerical simulation by means of cellular automata. The solidification process of molten steel and the heat exchange between the steel strip/air, the coating, the rolls and the cooling water in the channel of roll sleeves were simulated. The simulation results show that increasing the intensity of cooling water favors the nucleation and growth of columnar crystals but has little effect on the equiaxed crystals. Forced convection increases the degree of subcooling close to the coating position, which ultimately led to more grain formation, as shown in Figure 6 [41]. These predicted results were helpful to optimize casting parameters and improve the strip quality in the twin-roll strip casting process.

**Figure 7:**
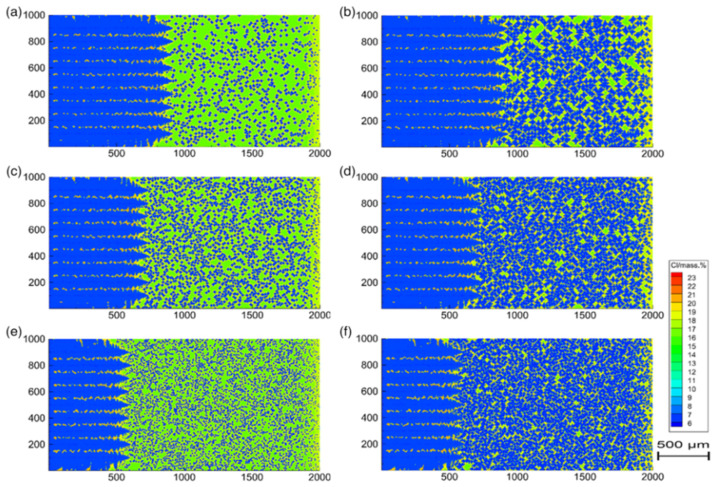
The solute concentration distributions of dendrite growth under different vibrations. (**a**–**f**) 400, 500, 600 Hz, 0.2 mm, ΔX¼ 1 μm, 1000 × 1000 meshes, respectively [42].

Vibration-excited liquid metal nucleation is a solidification microstructure control technology that promotes the growth of equiaxial crystals by vibrationally chilling the nucleation generator. However, due to the high crystallization temperature of ferritic stainless steel and the surface of the nucleus generator rapidly forming a solidification shell because of the effect of excited cooling, the difficulty of real-time observation of the solidification process undoubtedly increases. Wang et al. [42] established a 2-D CA model of dendrite growth to simulate the solidification microstructure of Cr17 stainless steel processed by the vibration-excited liquid metal nucleation. The simulation results show that with the increase in vibration intensity, the temperature field distribution became more uniform, the area of the equiaxial dendrite zone was significantly enlarged, the grains were refined and the microstructure became more uniform, as shown in Figure 7 [42]. Furthermore, the formation of the solidified shell on the crystal nucleus generator surface was delayed.

For the past few years, researchers have added the finite element analysis (FEA) tool to the CA method, creating a tight coupling between the CA method and the FEA tool to simulate the solidification process of aluminum alloy under casting process conditions. This CA model could predict location-specific microstructures, including the secondary dendrite arm spacing and porosity [43]. In addition, some scholars have combined the CA method with the finite difference method (FDM) to establish a two-dimensional (2-D) multi-component and multi-phase CA model. This 2-D CA model could be used for simulating the evolution of gas microporosity and microstructure of ternary hypoeutectic Al-Si-Mg alloys, involving dendrites and irregular binary and ternary eutectics. Furthermore, this 2-D CA model provided an effective method to study the mechanism of porosity formation, dendrites and irregular ternary eutectics growth during the solidification of ternary Al-based alloy [44]. 

**Figure 8:**
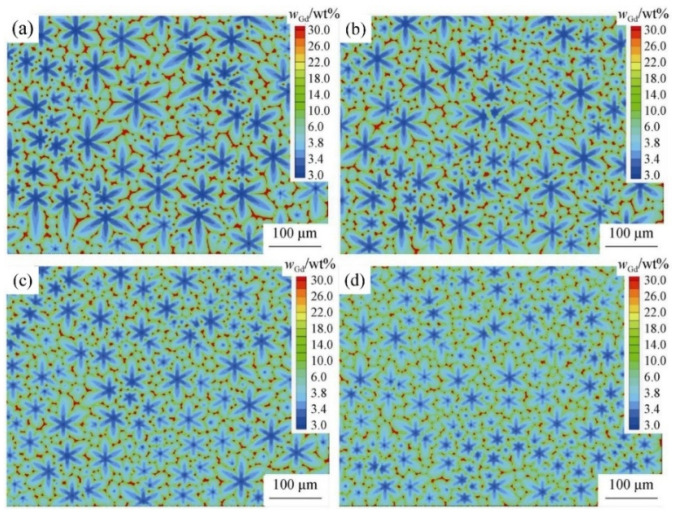
Simulation results of Mg-Gd-Y-Zr alloy with 0.58 wt% Zr at various cooling rates. (**a**) 2.6 °C·s^−1^, (**b**) 3.3 °C·s^−1^, (**c**) 4.4 °C·s^−1^, (**d**) 6.1 °C·s^−1^ [46].

Due to the large size of the ingot, the crystallization process is slow, resulting in the solidification microstructure that is very coarse and uneven. In order to solve this problem, researchers have simulated the formation and growth of solidification columnar crystal by the CA model and adjusted the process parameters to realize grain refinement and homogenization. For the purpose of exploring the evolution law of microstructures in a non-uniform temperature field, including dendrite morphology and tip growth rate, some scholars established a dendrite solidification CA model and compared differences and similarities between microstructure evolution under uniform and non-uniform temperature fields [45]. Besides, Wang et al. [46] investigated the solidification microstructure of Mg-Gd-Y-Zr alloy to study the effects of the cooling rate and Zr content on the grain size via an experimental study and CA simulation. The results showed that the grain size decreased with an increase in the cooling rate and Zr content. Figure 8 shows the simulation results for the solidification microstructure of the Mg-Gd-Y-Zr alloy with 0.58 wt% Zr at various cooling rates [46].

**Figure 9:**
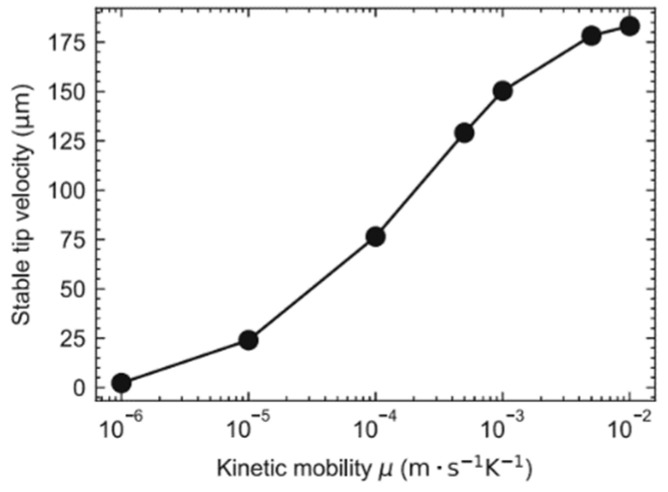
The stable tip velocity in the simulations with different kinetic mobility values [47].

Most cellular automata models assume local equilibrium at the interface, but this is no longer applicable to rapid solidification with high supercooling encountered in casting, especially welding and additive manufacturing. Therefore, Liang et al. [47] established a new non-equilibrium CA model. The kinetic subcooling and non-equilibrium effects at the interface have been taken into account, and a new diffusion term has been proposed to deal with the diffusion between the interfacial and liquid units in the CA model. The established CA model was used to simulate the solidification model of single dendrite growth of Al-3Cu alloy under different supercooling conditions. The results show that the stabilized tip velocity increased and tended to converge as the kinetic mobility increased, as shown in Figure 9 [47].

**Figure 10:**
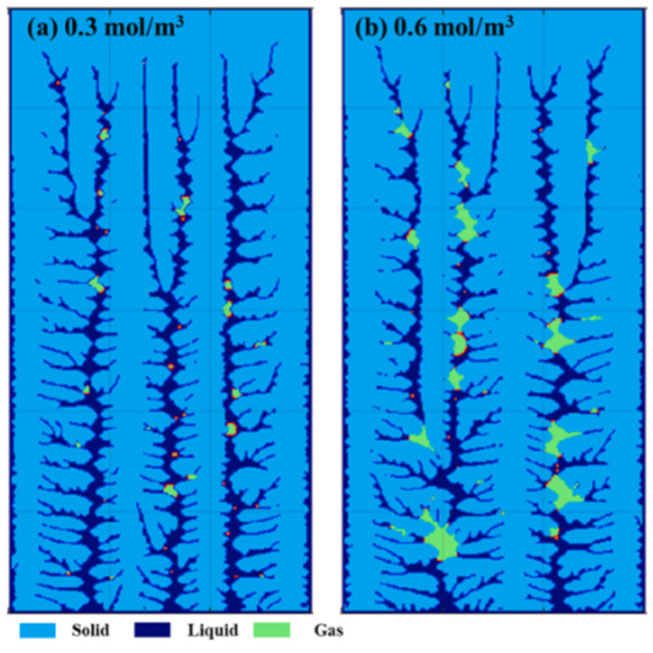
Columnar dendrite of cooling rate of 12 K/s with respect to the initial hydrogen concentration: (**a**) 0.3 mol/m^3^, (**b**) 0.6 mol/m^3^, liquid phase (deep blue), solid phase (light blue), gas phase (green) [48].

The lattice Boltzmann method (LBM) is a mesoscopic computational fluid dynamics approach favored for its simplicity in describing fluid interactions and ease in handling complex boundary conditions. Moreover, LBM is particularly suitable for simulating nano/microscale flows, mass transfer issues and the transport of rarefied gases because it is not constrained by the continuum assumption. However, the solidification process is a complex phenomenon involving multiple scales from the microscopic to the macroscopic. Therefore, to enhance the accuracy and reliability of simulations, it may be necessary to combine LBM with other simulation methods such as the CA method to effectively address the challenges of multi-scale modeling. Lee et al. [48] made an attempt to construct a lattice Boltzmann method-cellular automata (LBM-CA) model to predict the porosity evolution and dendritic growth in the solidified Al-Cu alloy. The morphology of the microstructure, including gas porosity and solid dendrites, mainly depended on the cooling rate and the initial hydrogen concentration in the melt. The results show that the percentage of porosity increased at lower cooling rates and the pore size also increased at higher hydrogen concentrations, as shown in Figure 10 [48]. The model could be used to optimize solidification conditions to reduce porosity defects in solidified materials, including casting processes and welding processes as well as 3-D printing.

**Figure 11:**
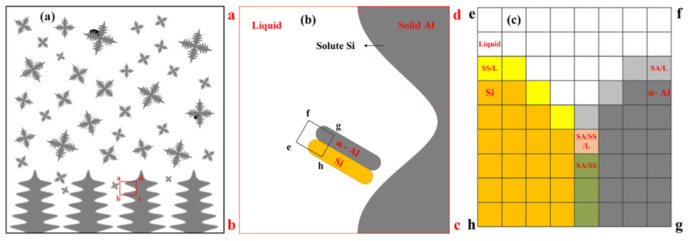
Schematic diagram of solidification dendrites and eutectic phases. (**a**) Dendrite morphology during solidification in mesoscale; (**b**) eutectic phase near dendrite squared corresponds to the red square in (**a**); (**c**) the variable of the state in this CA model corresponds to the black square in (**b**). The yellow color represents the Si phase, while the gray color represents the α-Al phase [49].

The morphology of eutectic Si in solidified microstructures is critical to the properties of Al-Si-based alloys. Simulating the formation of the eutectic Si phase is a challenge in the design and fabrication of solidification products of Al-Si based alloys based on integrated computational materials engineering. Gu et al. [49] developed a multicomponent 3-D CA model including the nucleation and evolution of the eutectic transition at the end of solidification. Figure 11 shows the schematic diagram of solidification dendrites and eutectics in this model [49]. The method was able to simulate the evolution of the whole solidification process, including dendrite growth and eutectic transformation during casting, welding and additive manufacturing.

#### 3.1.2. CA Simulation in Welding

Welding is one of the common joining techniques in metal processing. Complex processes such as material melting, solidification and crystal growth during welding can be simulated using CA to study the quality of welds and the factors affecting them.

**Figure 12:**
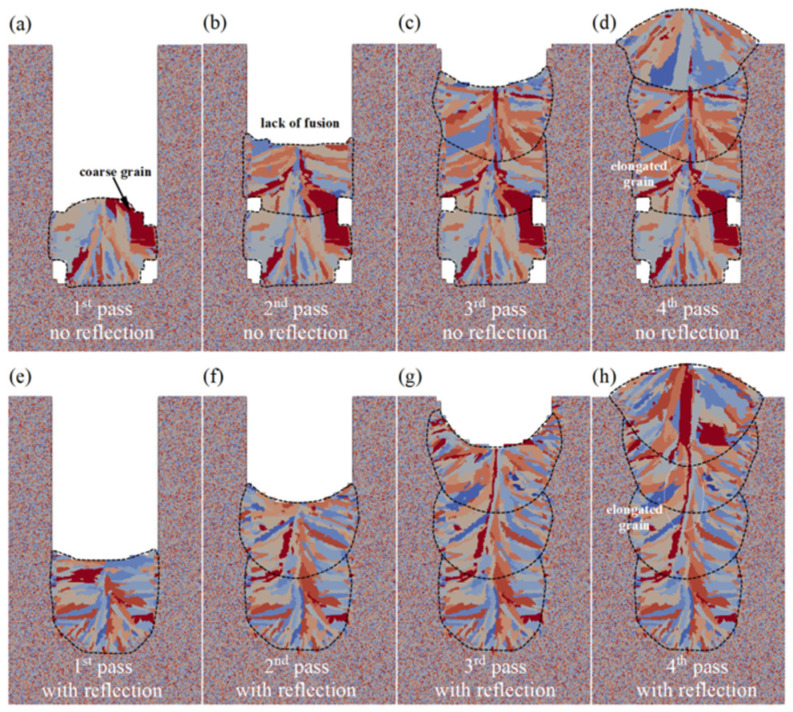
Grain structure evolution of four welding passes without laser beam reflection: (**a**–**d**); and with laser beam reflection: (**e**–**h**). The white areas represent lack of fusion, while other different colors represent different grains [50].

In laser welding processes such as narrow gap laser welding (NGLW), the laser beam interacts with the sidewall several times before forming a narrow groove, which has a strong influence on the formation of defects and the development of the grain structure. Gu et al. [50] established an integrated ray tracing-based computational fluid dynamics (CFD) and CA modelling framework to investigate the impact of laser beam reflection on molten pool formation and microstructure evolution in multi-pass NGLW. Figure 12 illustrates the comparison of microstructures on transverse cross-sections in the middle of the welded bead after solidification, with and without laser beam reflection. Finally, the simulated molten pool morphology and grain structure were compared with experimental observations, and good agreement was achieved [50].

**Figure 13:**
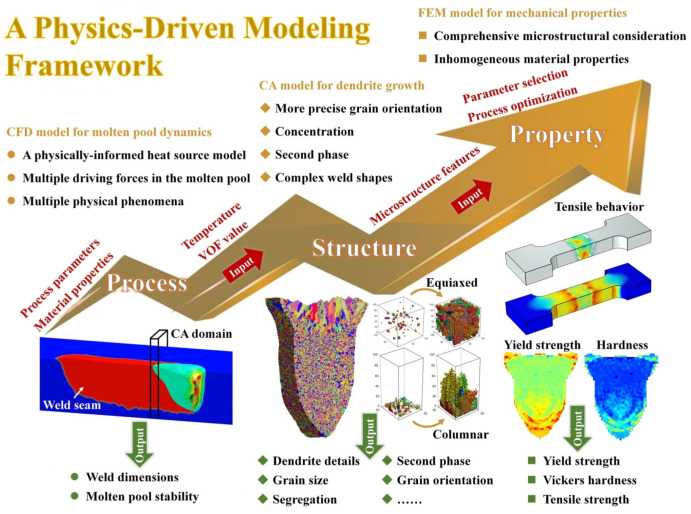
Schematic of the modeling framework to predict the mechanical properties of the welded joints by electron beam welding (EBW). The numerical method and advantages of each model are specified [51].

The process–structure–property relationship has been the focus of research in the field of welding. Yang et al. [51] developed an integrated model to reproduce the complex melting and solidification processes in the electron beam welding of Al-Cu alloys by using the computational fluid dynamics (CFD) model to calculate the fluid dynamics, the CA model to simulate the dendrite growth and the FEM model to predict the tensile strength. The integrated modeling framework is shown in Figure 13 [51]. The combination of process–structure–property was realized, which helped to optimize the actual welding process to customize the microstructure and mechanical properties.

**Figure 14:**
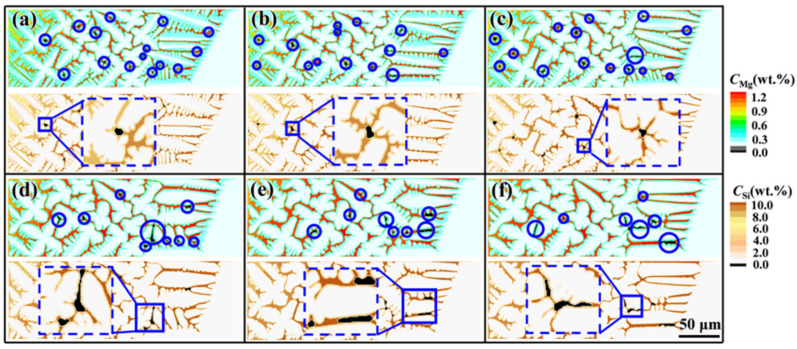
Simulated morphologies of welding porosities and dendrites under different heat inputs when the solid fraction reaches 0.85, shown in the Mg and Si concentration fields: (**a**) 200 J/mm; (**b**) 250 J/mm; (**c**) 300 J/mm; (**d**) 400 J/mm; (**e**) 500 J/mm; (**f**) 600 J/mm. The blue circles in the panels of the Mg concentration field indicate the gas pores. Each dashed square is a local magnification of the corresponding solid square in the Si concentration field [52].

Chen et al. [52] developed a cellular automaton-finite difference-lattice Boltzmann (CA-FD-LB) model to simulate weld porosity formation and dendrite growth during the welding of Al-Mg-Si alloys. The effects of heat input on weld porosity and dendrite morphology as well as porosity when changing the welding power were analyzed. As the heat input decreased, the dendrite arms became finer, the percentage of porosity decreased, and the number of gas pores increased, as shown in Figure 14 [52]. The model also guided the prediction of the microstructure of welded joints. 

#### 3.1.3. CA Simulation in Additive Manufacturing

Additive manufacturing represented by 3D printing, as a new material forming technology, has developed rapidly in recent years [53]. Additive manufacturing technology can be divided into electron beam selective melting (EBSM), direct laser deposition (DLD), laser powder bed fusion (LPBF), selective laser melting (SLM) and so on, according to the different heat sources and material. To date, numerical simulation has been a powerful tool to understand the complex physical processes occurring in the metal additive manufacturing process and provide guidance for the optimization of process conditions [54]. 3-D printing must involve rapid solidification, liquid–solid phase transformation, formation and evolution of microstructure, etc., which are suitable to deal with metal physics problems via CA. Furthermore, CA have also made breakthroughs in metal additive manufacturing technology in the last five years [55,56,57]. 

**Figure 15:**
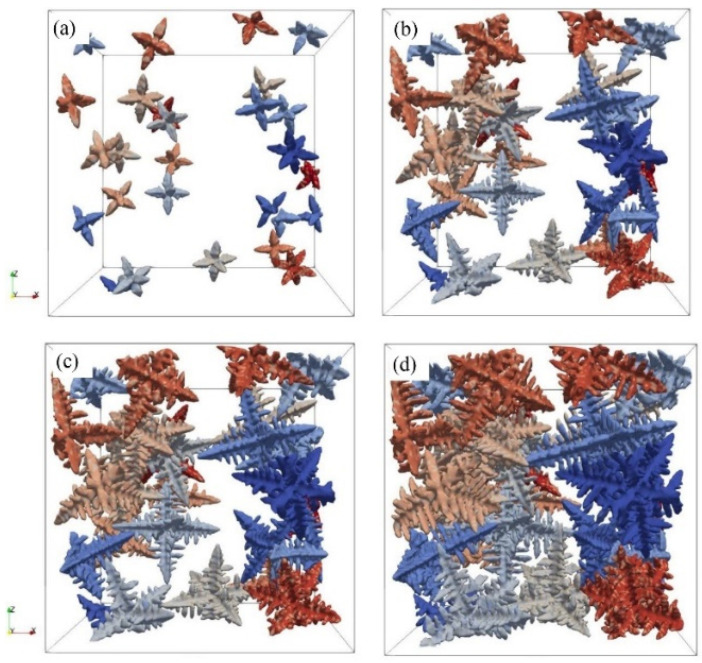
Simulation results of multi-dendrite growth in chronological order: (**a**) 0.008 s; (**b**) 0.016 s; (**c**) 0.020 s; (**d**) 0.028 s. Different colors represent different dendrites [55].

Yu et al. [55] used the CA method to study the influence of different additive manufacturing technologies on the evolution of materials solidification processes. As for electron beam melting technology, a multi-grid CA model for the dendrite growth was developed, which was applied to vividly reproduce the dendrite growth of nickel-based superalloy during the process of single-track EBSM. Moreover, Figure 15 shows the simulated dendrite morphology in chronological order, and this CA model successfully presented dendritic growth in random orientation [55].

**Figure 16:**
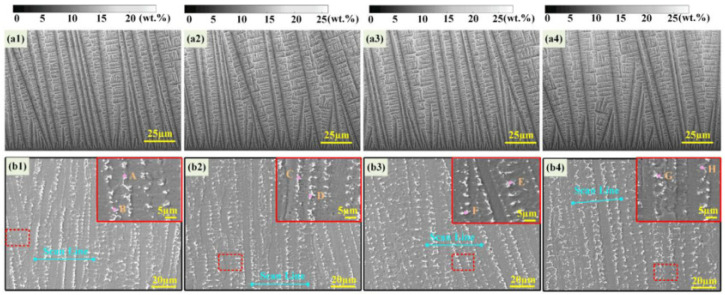
Comparison of dendrite simulation and experiment under different laser powers: (**a1**) simulation results of 800 W; (**a2**) simulation results of 1000 W; (**a3**) simulation results of 1200 W; (**a4**) simulation results of 1400 W; (**b1**) experimental results of 800 W; (**b2**) experimental results of 1000 W; (**b3**) experimental results of 1200 W; (**b4**) experimental results of 1400 W. Nb element concentration analysis was conducted at various points from A to H. Each solid square is a local magnification of the corresponding dashed square in the experimental image of the dendrite and blue arrows represent scan lines [56].

Recently, Meng et al. [56] combined the finite element method (FEM) and CA method to simulate the solidification microstructure evolution of Inconel718 with different process parameters during the direct laser deposition (DLD) process [56]. The morphology and size of various microstructures for different laser deposition processes were discussed by this model. The results showed that with the increase in laser power, the primary dendrite arm spacing increased, as shown in Figure 16 [56].

**Figure 17:**
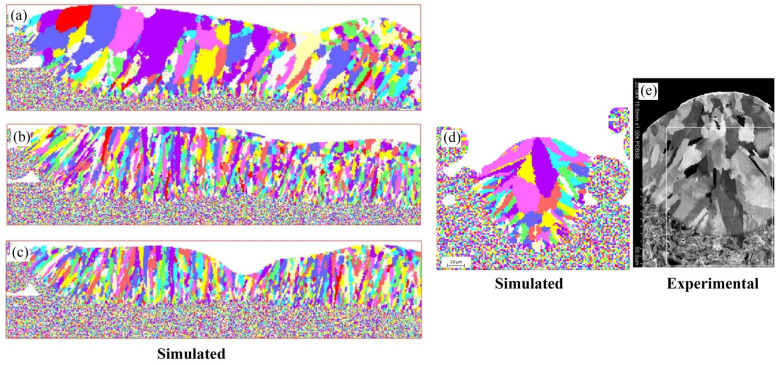
The longitudinal cross-sectional view of microstructures simulated by CA at various scan speeds: (**a**) 0.12 m/s; (**b**) 0.20 m/s; (**c**) 0.28 m/s [57]. The CA simulated cross-sectional images of the solidified grain structure at a scan speed of 0.12 m/s perpendicular to the laser moving direction, which were compared with the experimentally measured cross-sectional images from the literature [58]: (**d**) simulated [57]; (**e**) experimental [58]. White color shows air, and the other different colors represent grains with different orientations.

Moreover, Zhang et al. [57] established a new model by combining computational fluid dynamics (CFD) with CA to reproduce, and even predict, the solidification microstructure evolution of 316 L stainless steel during the laser powder bed fusion (LPBF) process. The simulation results showed that with the increase in laser scanning speed, the laser–grain angle and the columnar grain contents were growing, while the grain size was finer, as shown in Figure 17a–c [57]. The CA model simulations of solidified grain structure were compared with an experimental cross-section view of the solidified grain structure at 0.12 m/s scan speed [58] in order to verify the accuracy of the CA model simulations of solidified grain structures, as shown in Figure 17d,e [57]. A small region of equiaxed grains at the top of the melt pool was observed in the experimental results, which were formed by volume nucleation [59]. However, the CA model in this work could not capture the equiaxed grains initiated by volume nucleation, which showed a difference at the top region of the melt pool compared with the experiment [57].

In conclusion, by using the CA method, researchers have not only simulated the solidification microstructure evolution of metallic materials to guide the selection of process parameters, which can obtain the ideal microstructure, but have also compared the experimental study with the simulation study and verified the accuracy of the simulation results through the experiment.

### 3.2. Application of CA in Recrystallization Simulation

In 1991, Hesselbarth and Gobel [60] first proposed the application of CA in the research of recrystallization simulation. They used the CA model to study the kinetic simulation of recrystallization nucleation and grain growth under different model parameters and algorithms. Under the same model assumptions, they obtained the same results as the Johnson–Mehl–Avrami–Kolmogorov (JMAK) theory. Goetz et al. [61,62] further developed the above model and studied dynamic and static recrystallization, simulating the effects of different types of nucleation and nucleation densities on recrystallization kinetics. The above studies initiated and developed the application of the CA method in recrystallization microstructure simulation, making materials science scholars see the feasibility of the CA method in recrystallization microstructure simulation, which attracted more scholars to join in the research of using CA to reproduce the evolution of recrystallization microstructures. Recently, a large number of scholars have stood on the shoulders of giants and carried out many new studies around the simulation of recrystallized microstructure evolution by the CA method, including dynamic recrystallization (DRX) occurring during deformation and static recrystallization (SRX) occurring during annealing, etc.

#### 3.2.1. CA Simulation to DRX in Thermal Deformation

Dynamic recrystallization (DRX) is a significant improvement method during thermoplastic deformation processes, which mainly aims at material softening, grain refinement, microstructure control and plastic-forming ability. Moreover, the microstructure formed by DRX directly determines the mechanical properties of the material [63]. Domestic and foreign scholars have conducted a large amount of research on multiple research directions of DRX by using the CA method and have gained innovative results, including using a constitutive model and the CA method to simulate microstructure evolution [4,64].

In the studies of CA simulation of DRX microstructure during thermal deformation, the vast majority of dislocation mechanisms are used to model dynamic recrystallization of metal-forming processes [65,66,67,68]. During thermal deformation, the dislocation density is increased with the increase in strain; the stored energy after deformation becomes the nucleation driving force for recrystallization. Moreover, the nucleation of dynamic recrystallization is related to the accumulation of dislocation density. The essence of grain growth is the migration of grain boundaries, and the driving force for grain boundary motion is the interfacial energy provided through the curvature of the grain boundary. Furthermore, the nucleation of recrystallized grains starts at grain boundaries only when the dislocation density of the heat-deformed metal reaches a critical value. The new grains consume the dislocation density and bring it back to its pre-deformed value, and then the new grains continue to grow at a certain rate; the dislocation density of the new grains increases with the increase in strain. Grain growth stops when the grain growth drive decreases to zero or when recrystallized grains collide with other nascent grains. Meanwhile, the flow stress varies with the dislocation density during the dynamic recrystallization process. 

Therefore, CA is used to simulate the microstructure evolution of dynamic recrystallization during thermal deformation, while also obtaining the variation of the dislocation density field. More importantly, the flow stress curve can be obtained by simulation, which makes the use of CA methods more progressive than microstructure simulation for the prediction of mechanical properties. 

**Figure 18:**
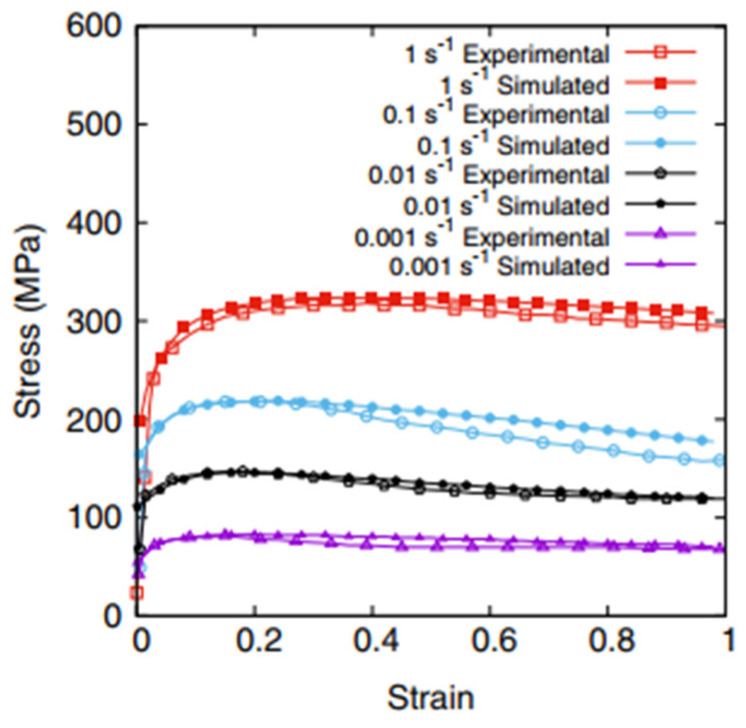
Comparison of flow stress response at temperature 1040 °C and various strain rates [65].

**Figure 19:**
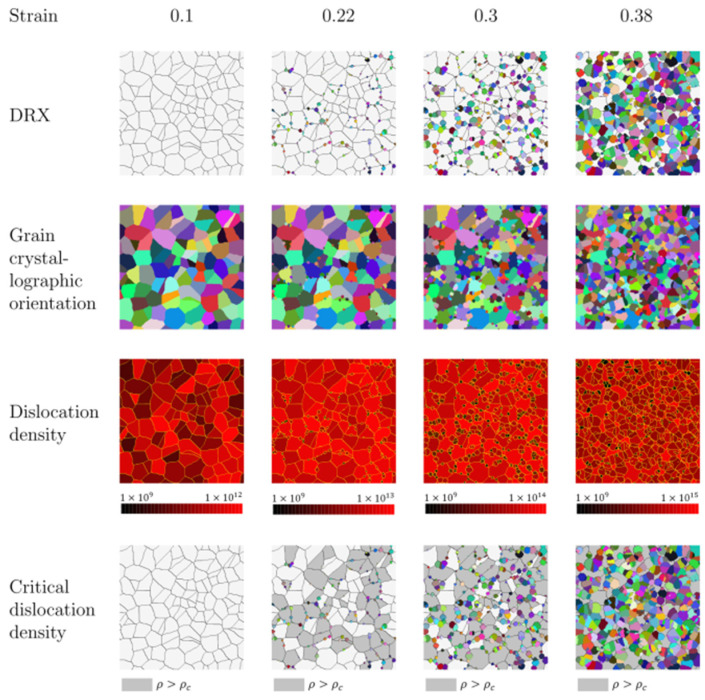
Simulation results for strain rate 0.01 s^−1^ at deformation temperature 1040 °C in the case of random orientations. The depth of red color represents the change of dislocation density, gray color represents that the dislocation density of grains is greater than the critical dislocation density, and other different colors represent different grains [65].

Alone et al. [65] have studied dynamic recrystallization (DRX) in a Ni alloy at deformation temperature 1040 °C and different strain rates using a cellular automata model. The evolution of dislocation density was based on the Kocks–Mecking equation. Nucleation occurred at grain boundaries when the dislocation densities in neighboring grains exceeded a threshold value. Moreover, a new methodology for nucleation probability was developed to predict the number of active nucleation sites at each marching time interval based on the deformation temperature and strain rate. The grain growth model was based on driving force and grain boundary mobility, which were functions of the grain boundary energy and misorientation between neighboring grains, respectively. The CA model was able to predict the flow stress response with greater accuracy at relatively low strain rates, as was apparent from the decreased deviation between the simulated and experimental curves, as shown in Figure 18 [65]. The simulated results of DRX microstructure and changes in dislocation density for the strain rate 0.01 s^−1^ at the strains 0.1, 0.22, 0.3 and 0.38, respectively, at 1040 °C are shown in Figure 19 [65]. All nuclei are represented with different colors as random orientations have been assigned. Nucleation at grain boundaries was observed post a critical dislocation density. This study could help in designing thermo-mechanical treatment and metal-forming process solutions to achieve customized mechanical properties for specific applications. 

**Figure 20:**
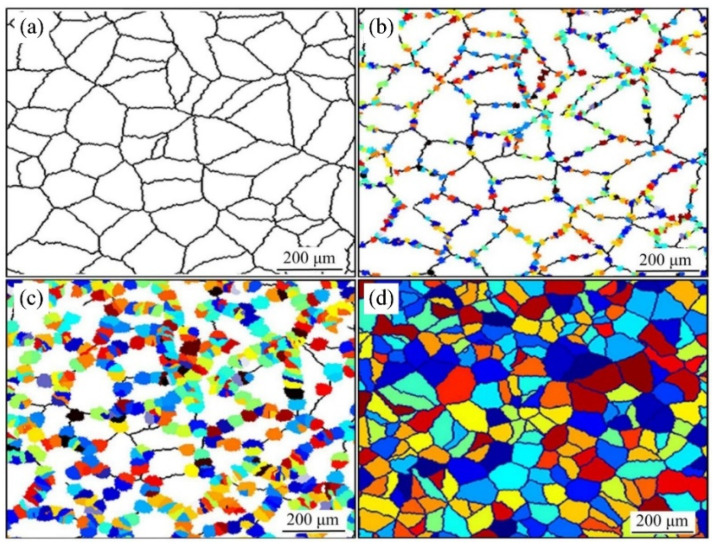
Predicted microstructure of V-10Cr-5Ti alloy at different strains: (**a**) 0; (**b**) 0.1; (**c**) 0.3; (**d**) 0.8. The state variable of colors is set by 0−255 for visualization. Different colors represent different grains [69].

Based on the Arrhenius model, Cao et al. [69] established a CA model of microstructure evolution of DRX processes in thermal deformation of V-10Cr-5Ti alloy. The CA model revealed the impact of thermal deformation parameters such as strain rate and deformation temperature on the microstructure evolution of DRX process. According to the DRX process of V-10Cr-5Ti alloy during hot compression deformation, as shown in Figure 20 [69], it was found that the process of DRX could be divided into three parts, including nucleation, grain growth and grain impingement. Moreover, the recrystallization fraction increased with the increase in strain, reaching 100% at the strain of 0.8, as shown in Figure 20d. 

Some scholars have simulated and analyzed the DRX process of different metallic materials under different deformation conditions by using the CA method. During a double-pass continuous expansion extrusion-forming process, the grain refinement mechanism of Al-Mg-Si alloy rods was investigated by establishing a CA model. Furthermore, it was observed that the grain size of Al-Mg-Si alloy rods was remarkably refined after continuous dynamic recrystallization (CDRX) and geometric dynamic recrystallization (GDRX) [70]. Aiming at the grinding strengthening process, the researchers established a CA model to simulate the microstructure evolution of the grinding enhancement layer of 40Cr alloy steel during DRX processes due to the severe plastic deformation at high temperature [71]. 

In addition, based on the CA method, scholars have also investigated microstructure evolution of DRX of some other alloy materials under different processing conditions, such as extrusion process of AZ80A magnesium alloy [72], abrasive grinding hardening of 45# steel [73] and the hot isothermal forging process of TB8 titanium alloy [74]. These CA models of dynamic recrystallization of various metals were established, and the simulation results were essentially in agreement with the experimental results, which proved the accuracy and effectiveness of each model. These models could also provide guidance for the formulation of forming processes.

#### 3.2.2. CA Simulation of SRX in Annealing 

Static recrystallization (SRX) is an important means to change the microstructure of metallic materials during annealing, which can provide ideas for the control of microstructure evolution and then realize the regulation of mechanical properties. Many scholars have conducted a large amount of research on SRX during annealing by using CA [75,76,77]. Among the numerous studies on SRX, the work by Asgharzadeh et al. [77] is particularly noteworthy as they utilized the CA approach to investigate not only recrystallization simulation but also texture simulation, which is an area where applications of CA are relatively scarce.

**Figure 21:**
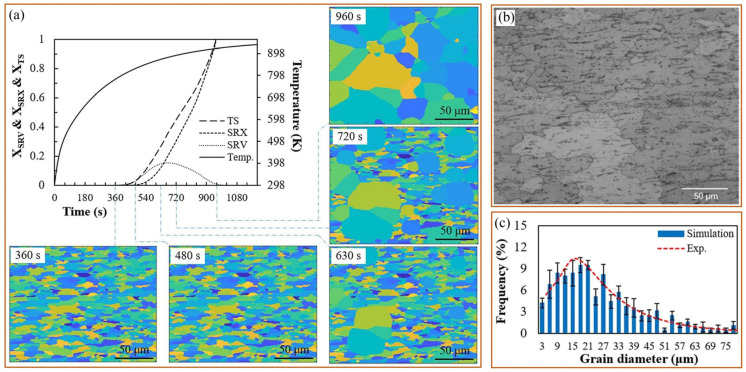
The simulation and experimental results for the samples deformed and annealed under path B at T = 960 K with a heating rate of h = 20 W/m·K: (**a**) the predicted softening fraction as well as the temporal microstructure evolution throughout the annealing of bulged specimen; (**b**) experimental result of fully recrystallized specimen; (**c**) comparison of experimental and predicted grain size distributions of fully recrystallized specimens. Different colors represent different grains [77].

**Figure 22:**
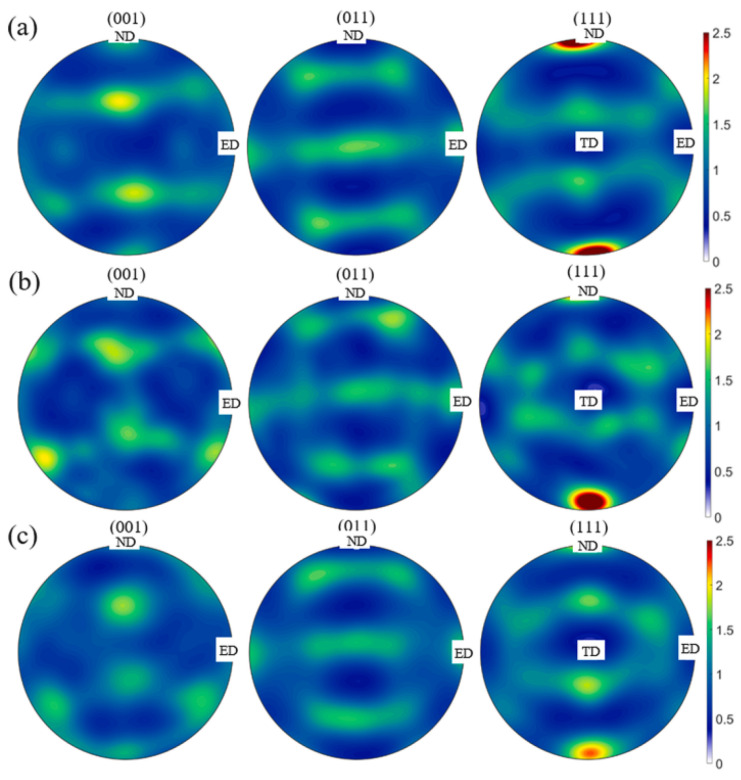
The predicted pole figures (PFs) for the bulged specimen deformed under path B and annealed at T = 960 K, h = 125 W/m·K and durations of 180 s, considering the recrystallization texture algorithms: (**a**) unchanged orientation; (**b**) average orientation; (**c**) random orientation [77].

Therefore, a hierarchically coupled CA model, crystal plasticity finite element method and thermal finite element model have been developed to predict the softening kinetics and recrystallization texture in non-isothermally annealed bulged tubes [77]. Using this model, the kinetics of softening mechanisms, including static recovery (SRV) and static recrystallization (SRX), were investigated, as shown in Figure 21a. Experimental results of a fully recrystallized sample are shown in Figure 21b, which were obtained from specimens deformed through path B and annealed at T = 960 K, h = 20 W/m·K. Comparing the simulated results in Figure 21a with the experimental result in Figure 21b, it becomes evident that the predicted grain topology was in close agreement with the experimental microstructure. To quantitatively demonstrate this concurrence, the corresponding grain size distributions of the microstructure are depicted in Figure 21c. It was found that the grain size distribution was precisely forecasted by CA with the data acquired through experimental observations. In addition, an algorithm based on the oriented nucleation theory was implemented into the CA model, through which the texture evolution during the progress of recrystallization was predicted, as shown in Figure 22 [77].

#### 3.2.3. CA Simulation in Asymmetrical Rolling

Asymmetrical shear rolling is beneficial to the grain refinement and homogenization of metal plate, so it is noteworthy that some researchers have studied recrystallization behavior during asymmetrical shear rolling via the CA method [78,79,80]. 

**Figure 23:**
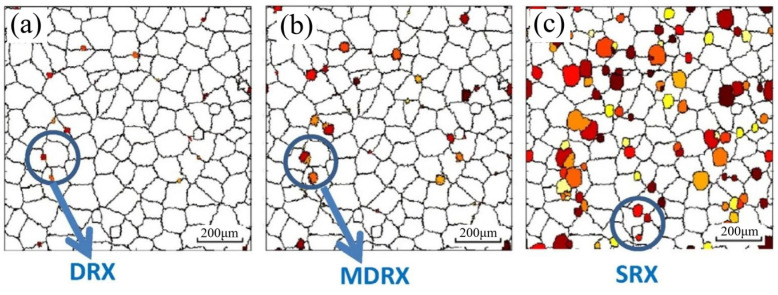
Microstructure variation at center point during 2nd pass and interval time: (**a**) t = 0 s; (**b**) t = 3 s; (**c**) t = 12 s. White regions represent the matrix and the color regions represent recrystallized grain [78].

By coupling finite element models (FEM) and the CA method, Zhang et al. [78] have realized the visualization of recrystallization during the asymmetrical shear rolling process. The coupling model was established to compare strain and temperature characteristics and microstructure evolution of the thick plate during multi-pass rolling of asymmetrical shear rolling and symmetrical rolling, respectively, in which different recrystallization mechanisms were taken into account, such as work hardening, dynamic recovery, dynamic recrystallization (DRX), meta-dynamic recrystallization (MDRX), static recovery and static recrystallization (SRX). Figure 23 shows microstructure variation at center point during 2nd pass and interval time, in which white regions represent the matrix and the color regions represent recrystallized grain [78]. 

Moreover, Sun et al. [79] established a DRX model-coupled macro-finite element and micro-crystal plasticity via the CA method, which realized the visualization of the DRX nucleation and growth of Mg-alloy sheet during the asymmetric warm-rolling process. Some scholars have combined experimental and CA simulation to explore the influence of several parameters of asymmetrical shear rolling such as speed ratio, temperature and rolling force on the recrystallization microstructure. Furthermore, in view of the influence of speed ratio, the CA model was established to analyze the effects of the offset distance and speed ratio on DRX fraction and grain size during the asymmetrical shear rolling process. The CA model comprehensively considered DRX of nucleation, grain growth and morphology [80]. 

In summary, based on the above study [78,79,80], we found that through the CA simulation and optimization method, the optimum parameter combination and process conditions in the process were explored to acquire superior rolling effect and mechanical properties. The models reproduced the recrystallization process and revealed the grain refinement mechanism of different alloys via the CA method, which provided new thoughts and methods for the optimization of process parameters. 

### 3.3. Application of CA in Phase Transformation Simulation

It is known that the phase transformation process is complex and short. In order to accurately reflect the phase transformation process, researchers initially used in situ observation to characterize the process at high temperatures [81]. However, this characterization method cannot reveal the continuous microstructure evolution. It has been shown that the continuous microstructure evolution of the phase transformation can be reproduced visually by the CA method. In 1998, Kumar et al. [82] established a CA model of nucleation and early growth in the austenite–ferrite transformation. The relationship between the grain size of ferrite, transformation fraction and cooling rate was obtained. Since then, scholars have used CA simulations to study a variety of phase transformation processes, including ferrite phase transformation, bainite phase transformation and martensite deformation during metal cooling, austenite-reverted transformation in annealing processes, as well as solid-state phase transformation in additive manufacturing.

#### 3.3.1. CA Simulation in Cooling Process after Hot Deformation 

Much research has been carried out in CA simulation of ferrite, bainite and martensite phase transformations occurring during cold cooling of steel materials. Recent studies showed that scholars tended to combine the CA method and other simulation methods to dynamically reproduce the microstructure evolution during phase transformation [83,84,85]. 

**Figure 24:**
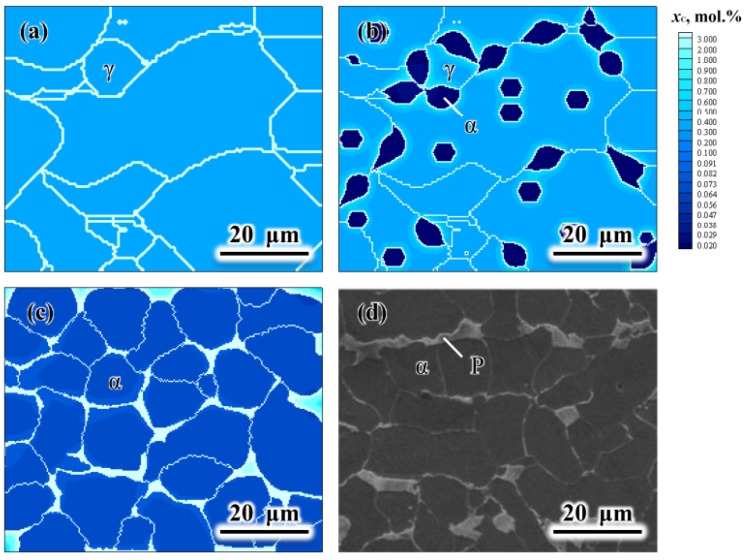
CA-simulated microstructure and carbon concentration fields during cooling from 832 °C to 650 °C at a cooling rate of 4 °C s^−1^: (**a**) T = 832 °C; (**b**) T = 750 °C; (**c**) T = 650 °C (t = 45.5 s, fα = ~0.92, Dα = ~9.4 µm); (**d**) SEM micrograph (fα = ~0.93, Dα = ~9.7 µm) of the cooled sample at room temperature [83].

Based on the FEM and CA models, Lin et al. [83] established a mesoscale CA model to study the microstructure evolution of the recrystallization and austenite (γ) to ferrite (α) transition of high-strength steels during a 6-pass hot-rolling process followed by air cooling. The CA model took into account the dislocation density, the carbon diffusion and the interaction between manganese atoms and α/γ interfaces. During cooling, the α-grains grew with the increasing carbon concentrations in both α- and γ-phases. The region near the α/γ interface was more carbon-rich due to the expulsion of carbon atoms from the newly formed α grains. When the simulation of phase transformation finished, the carbon content increased in the remaining γ-phase (light-blue in Figure 24), which corresponded to the subsequent pearlite phase. The CA model took into account the effect of manganese on the γ/α interface migration. The final α-fraction and the average α-grain size were simulated by the CA model, which were in great agreement with the experimental microstructure, as shown in Figure 24 [83]. 

**Figure 25:**
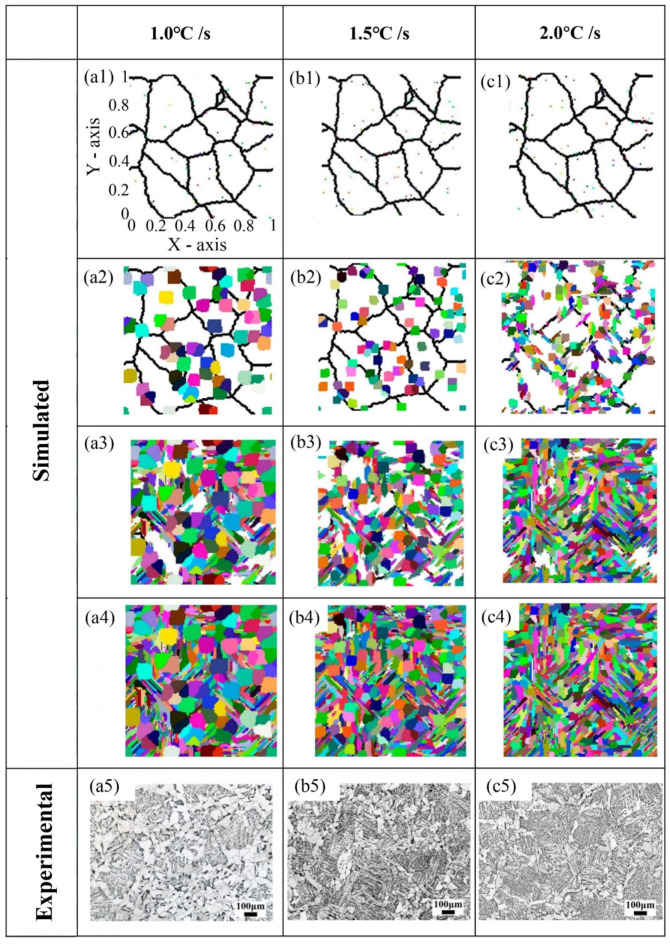
CA-simulated process of microstructure evolution with different cooling rates: (**a1**–**a4**) 1.0 °C/s; (**b1**–**b4**) 1.5 °C/s; (**c1**–**c4**) 2.0 °C/s; transformation fraction of (**a1**–**c1**) 0%; (**a2**–**c2**) 25%; (**a3**–**c3**) 50%; (**a4**–**c4**) 75% [84]. Experimental optical images characterizing the final microstructure achieved by different cooling rates: (**a5**) 1.0 °C/s; (**b5**) 1.5 °C/s; (**c5**) 2.0 °C/s [84].

In addition, math models such as Johnson–Mehl–Avrami–Kolmogorov (JMAK) theory play an important role in the simulation and prediction of phase transformation. Combined with the classic JMAK theory, Li et al. [84] simulated multi-phase transition kinetics of HSLA steel based on the CA method, and the microstructure evolution of the multi-phase transformation at different cooling rates was studied by CA. It was found that increasing the cooling rate could promote the nucleation of ferrite and bainite while also significantly refining the ferrite grains, as shown in Figure 25 [84]. In the simulation of microstructures, the black line represents grain boundaries of original austenite. The white zone denotes the untransformed austenite, and the colored zone denotes ferrite and bainite. The accuracy of the CA model was verified by comparing experimental and simulated results. As shown in Figure 25(a5–c5), the final microstructure of samples obtained from experiments conducted at different cooling rates demonstrated a marked reduction in the matrix area occupied by white polygonal ferrites as the cooling rate increased. Clearly, this experimental phenomenon was highly consistent with the simulated results.

The Lattice Boltzmann method (LBM) is a mesoscale simulation method that can simulate diffusion and heat flow during phase transitions and has been used in combination with the CA method to build new hybrid models in the last five years. Based on the CA and LBM methods, Łach et al. [85] developed a new hybrid model to study the diffusion phase transition of materials during heating, annealing or cooling. According to the simulation results acquired for one-dimensional modeling, the correctness of the interaction between LBM and CA in the general numerical solution was proven for the first time, as was the practicability of applying it to phase transformations modeling. Based on the above conclusions, the CA method has been combined with the LBM method to establish a three-dimensional heat flow model to simulate the heat transfer process during the diffusion phase transformation of carbon steel [86].

**Figure 26:**
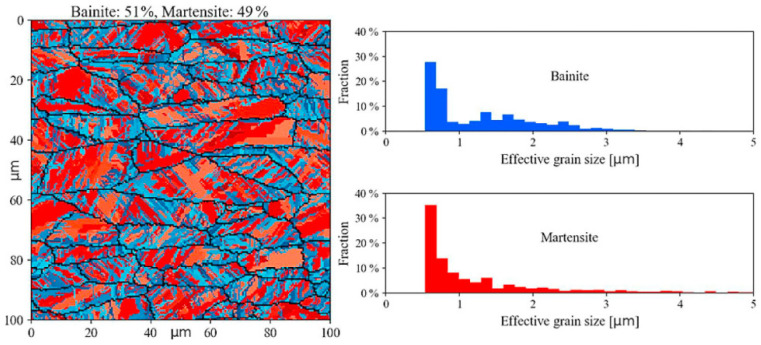
CA-simulated martensite and bainite growth in an austenitic grain structure and histograms of their effective grainsize. black lines are prior austenite grain boundaries, blues tones are bainite sheaves and red tones are martensite blocks [88].

In order to investigate the evolution law of martensitic transformation, Duan et al. [87] built a phase evolution CA model describing the microstructure of austenite martensitic transformation in high-speed dry cutting of steel GCr15. It is worth noting that during the high-speed dry cutting process of GCr15, the metamorphic layer with properties distinct from the base material is readily formed on the machined surface. The metamorphic layer is known as the ‘white layer’ due to its appearance under optical microscope, and austenite and martensite are important constituent phases in the evolution of white layer. This CA model simulated the phase transition of surface white layer and also studied the effect of different flank wear on the martensitic nucleation density. It was shown that reducing the flank wear could increase the number of martensite nucleations and enhance the strength of martensite in white layer on the machined surface to heighten the fatigue resistance of the machined surface. To develop a more detailed model for bainite and martensite transformations, Seppälä et al. [88] presented a novel two-dimensional cellular automata model for simulating the formation of lath martensite and bainite in steels during cooling. The results of this simulation could be used to estimate the fractions, shapes and sizes of bainite and martensite for different cooling rates, as shown in Figure 26, which should offer new possibilities for the qualitative estimation of the mechanical properties of high-strength steels with bainitic–martensitic microstructure formed from thermomechanically deformed austenite [88]. 

#### 3.3.2. CA Simulation in Austenite-Reverted Transformation in Intercritical Annealing

During the heating process of intercritical annealing of dual-phase (DP) steels, complex microstructure changes occur, involving austenite phase transformation and ferrite recrystallization, etc. Scholars have established a two-dimensional CA model to simulate the austenite-reversed transformation (ART) in the intercritical annealing process, which provided a means to analyze the laws and mechanisms of microstructure evolution of DP steels in the annealing process [89,90]. 

**Figure 27:**
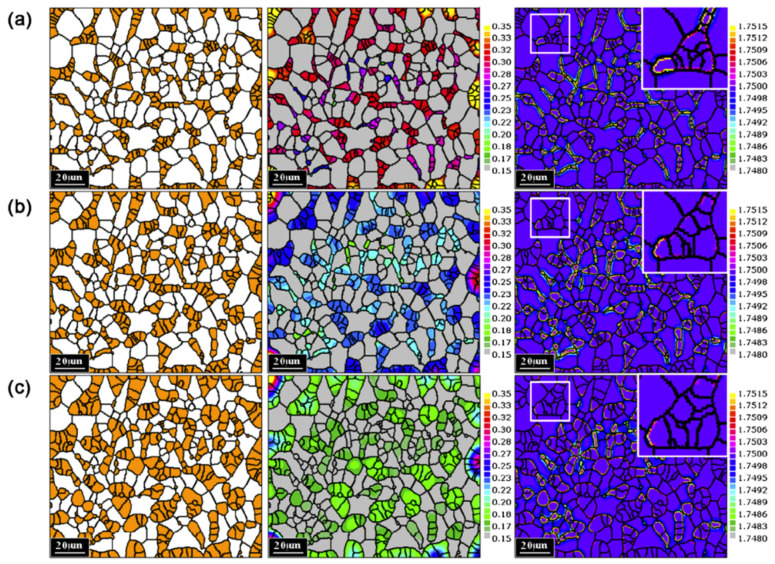
Simulation results of microstructure (left), carbon concentration field (middle) and manganese concentration field (right) when t = 75 s at different annealing temperatures in a Fe-0.08C-1.75 Mn (in wt.%) steel: (**a**) T = 740 °C; (**b**) T = 760 °C; (**c**) T = 780 °C. The yellow areas are the newly formed austenite, the white regions are ferrite phase and the black lines indicate the grain boundaries. Each square is a local magnification of the corresponding area in the manganese concentration field [91].

For the purpose of reproducing the austenite-reverted transformation process, Jia et al. [91] developed a mesoscopic CA model to describe austenite formation from ferrite-plus-pearlite microstructure of a C-Mn steel during intercritical annealing. This CA model described three-stage kinetics of the transformation combined with thermodynamic analysis, which made kinetics switching from fast austenite growth without Mn partitioning to sluggish austenite growth with Mn partitioning to bring off. The effect of the annealing temperature on the transformation kinetics and solute partition was also quantitatively rationalized using this model. Figure 27 [91] shows that increasing the annealing temperature was conducive to the austenite formation owing to the diffusional transformation nature. However, the Mn partitioning process might be delayed, depending on the driving force. In the microstructure, the yellow areas are the newly formed austenite, and the white regions are ferrite phase. The black lines indicate the grain boundaries. 

#### 3.3.3. CA Simulation in Solid-State Phase Transformation in Additive Manufacturing

**Figure 28:**
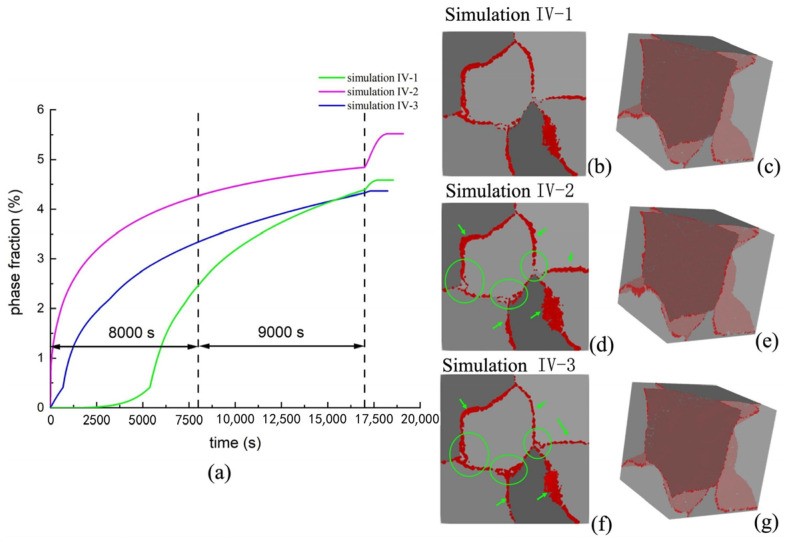
CA-simulated fractions and microstructure of the precipitates: (**a**) the fractions of the precipitates change over time; (**b**–**g**) the cross-sections and 3-D structures of the final microstructure of simulation IV (**b**,**c**), IV-2 (**d**,**e**) and IV-3 (**f**,**g**), respectively. Red clusters along the grain boundaries are precipitates. Green arrows indicate precipitates along the grain boundary and green circles indicate precipitates near the grain junctions [92].

Researchers have performed substantive simulation research in the field of additive manufacturing by using the CA method, especially with regard to metal solid-state phase transformation. Yu et al. [92] have used the CA method to investigate the evolution law of the phase transformation process of metallic materials in additive manufacturing. A mixed-mode phase transformation CA model was developed to simulate the solid-state phase transformation with alloy element partition. The CA model successfully reproduced the complex precipitation process of Al-Cu alloy during electron beam selective melting (EBSM), including the growth and decomposition of precipitates, as shown in Figure 28 [92].

**Figure 29:**
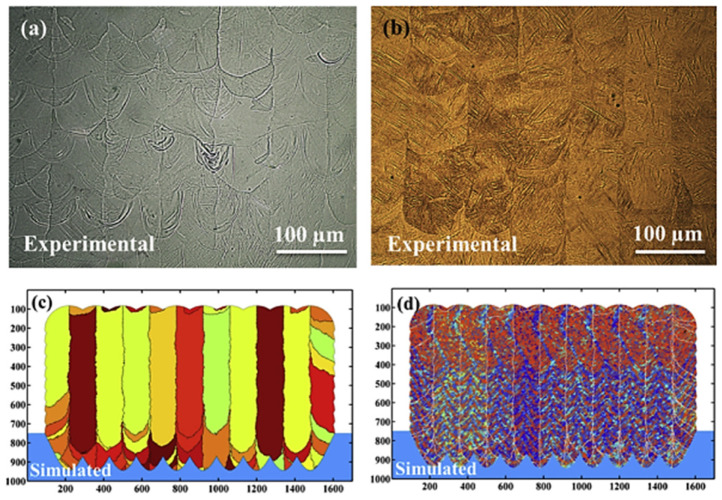
Comparison of experimental and CA-simulated microstructures of ten-track ten-layer samples: (**a**) experimental prior β grains; (**c**) simulated prior β grains; (**b**) experimental martensite within prior β grain boundaries; (**d**) simulated martensite within prior β grain boundaries, respectively. Different colors represent different grains [93].

Furthermore, in order to explore the microstructure evolution of solidification and solid-state phase transformation processes of Ti-6Al-4V alloy during selective laser melting (SLM), Yang et al. [93] used the CA method to simulate the mechanism of microstructure evolution under various spatially variable thermal cycles. The morphology and size of the β grain and martensite simulated by the model agree well with the experimental results in single-layer, thin-wall and multi-track multi-layer samples, as shown in Figure 29 [93]. According to the results of the microstructure evolution simulated by the two-dimensional CA model, the SLM deposition process of Ti-6Al-4V alloy was divided into three zones, including powder melting, remelting and reheating zones; and into four stages, including powder melting, mushy, multi-phase and solid-state phase transformation stages. As a result, the CA simulation results could provide improved guidance for the design of each additive manufacturing process parameter.

In brief, that scholars have used the CA method and other simulation methods to reproduce the complex microstructure evolution of phase transformation of various materials is a major breakthrough in the study of metallic materials phase transformation.

### 3.4. Application of CA in Carbide Precipitation Simulation

The strengthening method by second-phase particles is an important method of toughening metallic materials, where precipitates of complex-phase organization, interactions of hydrogen with second-phase particles and nanoparticle co-precipitation reinforcement have an impact on strength, toughness and other service properties of the material. The understanding of strengthening and toughening of second-phase particle is also growing, and in this area of research, scholars have attempted to use CA in simulation studies of carbide precipitation in addition to the need to rely on the fine characterization techniques of atomic-scale experiments [94,95,96,97,98].

#### 3.4.1. CA Simulation in Carbonitride Precipitation of High-Strength, Low-Alloy Steels

High-strength, low-alloy (HSLA) steels refer to the use of Nb, Ti, V and other micro-alloying elements produced by precipitation strengthening and grain refinement to obtain high strength of low-carbon steels. During thermomechanical rolling, carbon and nitrogen compounds can inhibit austenite grain growth, recrystallization and other processes. Accumulation of more deformation bands, dislocations and other ferrite nucleation sites in deformed austenite results in the refinement of ferrite grains. After final rolling, microalloying elements in solid solution in HSLA steels continue to precipitate during cooling, coiling, etc. The precipitation modes include precipitation during austenite cooling, interphase precipitation at the austenite–ferrite interface and uniform precipitation in ferrite. Recently, scholars have attempted to use CA to simulate carbonitride precipitation processes of HSLA steels [94,95,96].

**Figure 30:**
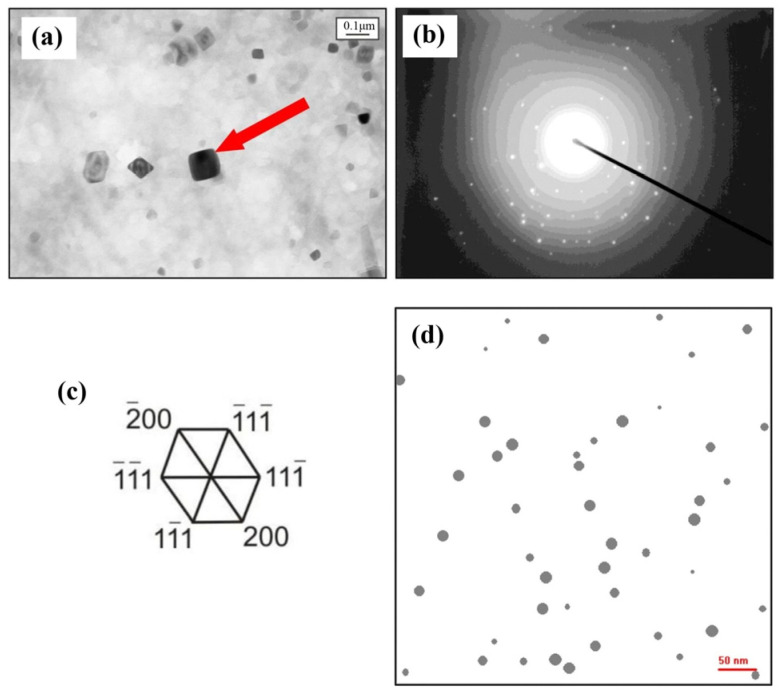
Experimental and simulated images of V(C,N) precipitations in steel (containing 0.3% C, 0.078% V 1.88% Cr and 0.0412% N) subjected to austenitization at 1200 °C, followed by holding at T = 850 °C for 20 h and quenching in water: (**a**) experimental microstructure, the red arrow represent the V(C,N) precipitation; (**b**) diffraction image of V(C,N); (**c**) solution of diffraction patterns (zone axis: [011] V(C,N); (**d**) simulated image [94].

**Figure 31:**
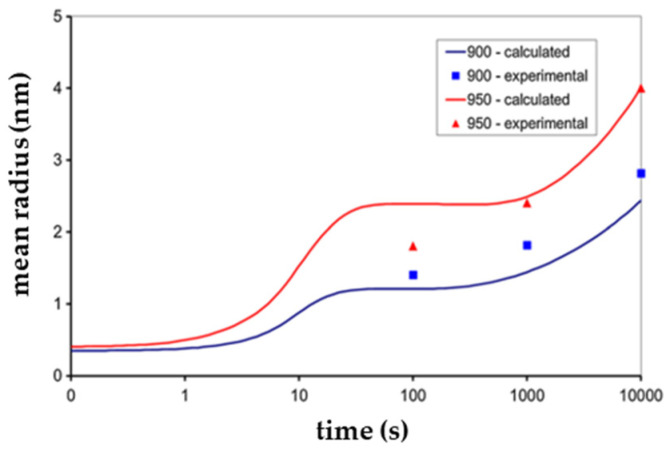
Comparison of the calculated with experimental data on mean radius of Nb(C,N) precipitation of steel containing 0.11% C, 0.03% Nb and 0.01% N at T = 900 °C and T = 950 °C for time = 10,000 s [94].

Marynowski et al. [94] presented a CA model of carbonitride precipitation to simulate images of microstructures in HSLA steels, and precipitates’ size (mean radius) and content (volume fraction) were obtained. Compared images of experimental and simulated images of V(C,N) precipitations showed satisfactory similarity, although the particle size of the simulated image was slightly smaller compared to real particles, as shown in Figure 30 [94]. At the same time, Figure 31 shows the comparison of calculated and experimental data of mean radius of Nb(C,N) precipitation [94]. Comparison of the simulated and experimental data showed a satisfactory convergence, which proved that the developed CA model could be a useful tool to support the design of heat treatment parameters for obtaining the desired mechanical properties of steel.

#### 3.4.2. CA Simulation in Carbonitride Precipitation of Fe-C-Cr Alloy

**Figure 32:**
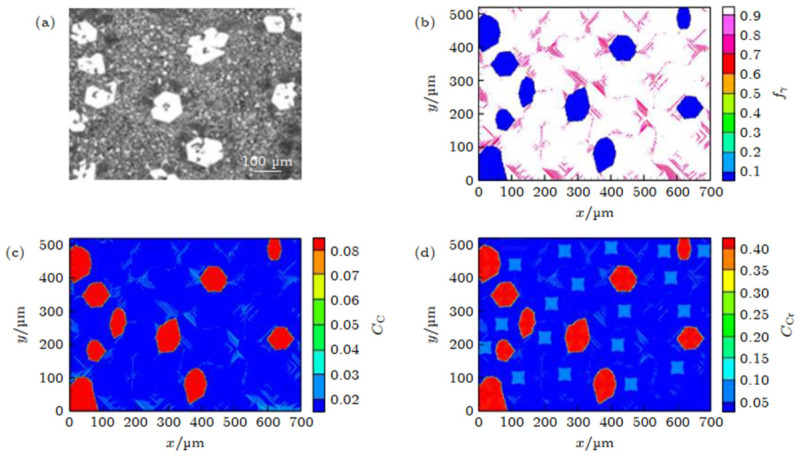
Experimental and simulated solidification microstructure for Fe-4%C-17%Cr alloy: (**a**) experimental microstructure; (**b**) simulated austenite mass fraction; (**c**) simulated C concentration field; (**d**) simulated Cr concentration field [97].

M_7_C_3_ carbides in high chromium cast irons are of moderate size and diffusely distributed, which is favorable to improve the wear resistance of the alloy. In order to analyze the morphology and distribution of M_7_C_3_ carbide grains in the matrix during solidification, the interaction between carbides and austenite grain growth and the total influence on the final M_7_C_3_ carbides’ size, Zhang et al. [97,98] built a two-dimensional microscopic CA model for the growth of the faceted M_7_C_3_ carbide together with the austenitic dendrite grains in an Fe-4%C-17%Cr ternary alloy. Figure 32 [97] shows the microstructure, simulated austenite mass fraction, C concentration field and Cr concentration field of Fe-4%C-17%Cr alloy under graphite-cooling conditions. The blue area is the M_7_C_3_ carbide grain, the white area is the austenite grain and the red area is the remaining liquid-phase region at the austenite arm. The volume fraction of M_7_C_3_ carbide in the experimental microstructure was 12.00%, as measured by Image-Pro Plus 6.0 software, and the volume fraction of M_7_C_3_ carbide in the simulation was 11.94%, which was similar to the experimental M_7_C_3_ carbide grain size and morphology.

### 3.5. Application of CA in Corrosion Simulation

For a long time, the corrosion and protection of metallic materials have been concerns of researchers. At present, the research methods of metal corrosion are still mainly experimental. Nevertheless, with the rapid development of computer technology, a growing number of scholars at home and abroad have combined numerical simulation technology, such as the CA method, with experimental laws as an accurate and intuitive research method for metal corrosion. Indeed, metal corrosion simulation can be considered a form of microstructure simulation as it involves microscopic processes such as chemical reactions, phase transformations and grain growth occurring at the material’s surface. These processes induce changes in the phase structure and microstructure, which subsequently affect the mechanical properties of metallic materials. Furthermore, the application of CA in the simulation of corrosion of metallic materials probably focuses on several aspects, including uniform corrosion, localized and pitting corrosion, intergranular corrosion, etc.

#### 3.5.1. CA Simulation in Uniform Corrosion

Uniform corrosion is a common type of metal corrosion characterized by the general occurrence of corrosion on the entire surface of the metal in contact with the corrosive medium. Furthermore, this type of corrosion leads to a continuous reduction in the cross-sectional area of metallic material, which negatively impacts its mechanical properties. Scholars have begun to apply the CA method to the simulation and study of the uniform corrosion process, thereby better revealing its corrosion mechanism and improving strategies for corrosion management. Wang et al. [99] developed a simplified CA model based on the stochastic approach through the reaction of diffusive corrosive gas and Ni-base alloy substrate in a chloride molten salt. Moreover, the CA model was adopted to simulate the growth of corrosion layers with the migration of the Cr element [99]. Based on the CA method, the corrosion process of 2195-T8 aluminum–lithium alloy in HNO_3_ was simulated in order to study the corrosion mechanism by using logistic distribution probability as a critical condition of corrosion probability [100]. Chen et al. [101] investigated corrosion behavior of marine structural steel in tidal zones based on wire beam electrode technology and partitioned cellular automata models. The CA model was adopted to simulate the initial corrosion evolutions of steel in the tidal zone. The proposed independent variable of the electrolyte concentration, corrosion probability, passivation probability and movement direction probability on the corrosion behavior of steel have been studied.

**Figure 33:**
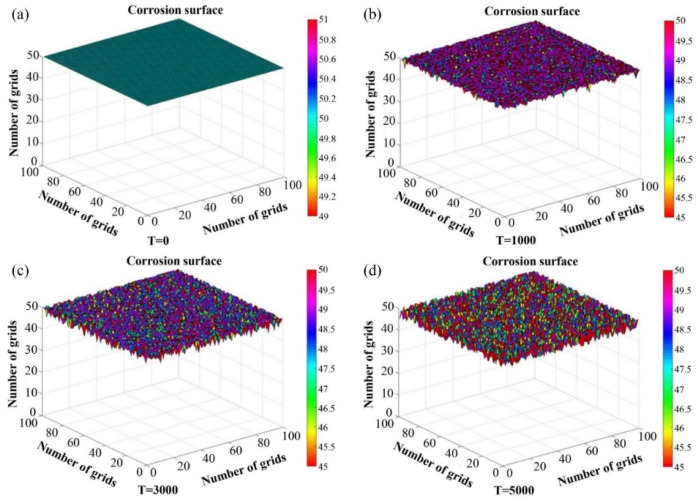
Schematic diagram of the change of outer corrosion layer with the increase in simulation time step T in the model: (**a**) 0; (**b**) 1000; (**c**) 3000; (**d**) 5000 [102].

Furthermore, for the purpose of investigating the corrosion behavior of nickel-based alloy in molten salt, Xu et al. [102] set the types and contents of different elements in a 3-D CA model and adjusted the program according to the corrosion mechanism. This CA model could be applied to predict the corrosion behavior in molten chloride salt of different metals, such as Hastelloy X and GH3535 alloys. Figure 33 [102] shows the variation of the outer corrosion layer with the increase in simulation time step in the 3-D CA model. Different colors represent different corrosion depths. It was found that the outer corrosion layer progressively formed and the thickness of the corrosion layer continued to grow with the increase in time step. The depth of corrosion of different positions on the corroded surface was simulated by the CA model, which could be used as a reference for the corrosion behavior of metals in molten chloride salt during the long-term service process.

#### 3.5.2. CA Simulation in Pitting Corrosion

**Figure 34:**
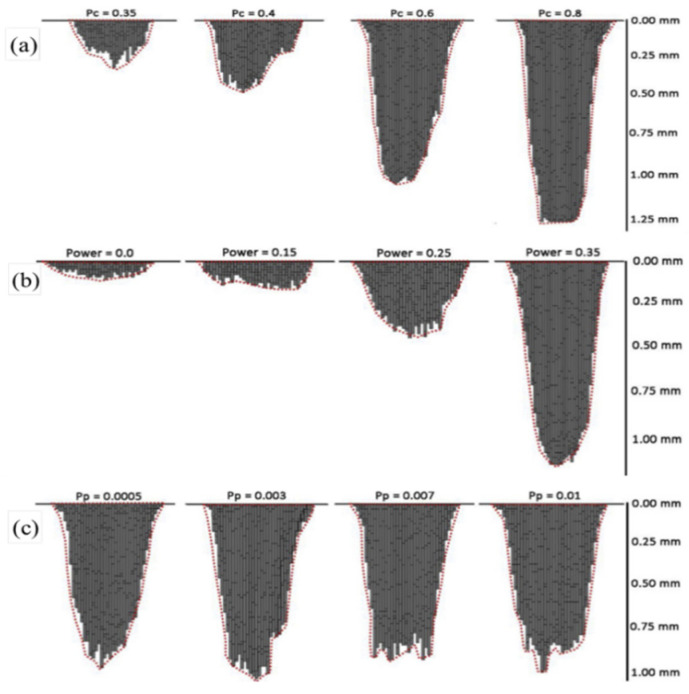
Simulated typical shapes of corrosion pits with different parameters: (**a**) corrosion reaction probability *Pc*; (**b**) probability of downward movement *Pd*; (**c**) passivation reaction probability *Pp* [107].

**Figure 35:**
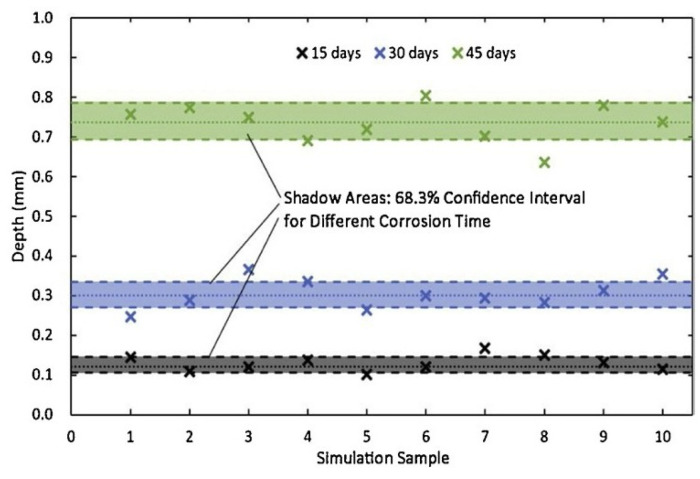
Comparison between logarithmic Gaussian distributions and CA simulation results. The dotted lines and shadow areas consist of the 68.3% confidence interval of the distribution model under different corrosion times, distinguishable by color. The scatter points refer to the depth results of ten CA simulations. The results better reflect both the uncertainty and rationality of the CA model [107].

Many metallic materials are sensitive to pitting corrosion, which can cause localized dissolution on the metal surface, resulting in small holes. Moreover, pitting corrosion often starts rapidly at the microscale and leads to failure of metallic materials at the macroscale. In order to reveal the microscopic dynamics of this complex process, the cellular automata (CA) method has been used to achieve a more precise understanding of the initiation and development of pitting corrosion and its relationship to the metal microstructure, thus providing the theoretical basis and experimental guidance for improving the pitting corrosion resistance of metallic materials. CA methods have been successfully used to analyze the mechanism of pitting corrosion [103,104,105,106]. In order to investigate the evolution laws of corrosion pits on Q345 steel surface under a salt-spray environment, Cui et al. [107] developed a 3-D CA model to reproduce the initiation process and growth process of corrosion pits simultaneously and to express the randomness of pitting evolution. Figure 34 shows that the maximum depths were controlled mainly by the corrosion reaction probability *Pc* and probability of downward movement *Pd*, whereas the pitting shapes were governed by the passivation reaction probability *Pp* [107]. To validate the accuracy of the CA model, it was challenging to obtain the above simulation results through experiments. Therefore, a mathematical model was developed based on the distribution laws of corrosion pits observed from experiments, which described the initiation and growth processes of corrosion pits. Figure 35 [107] shows the comparison between the mathematical model and the CA simulation results, revealing that the CA model exhibits a high degree of consistency with the mathematical model, which indicated that the CA model accurately reflected the conditions of corrosion pits. However, the CA model only predicted short-term growth laws of corrosion pits. How CA can be used for long-term prediction of corrosion pits is an issue that needs to be addressed in future research [107].

#### 3.5.3. CA Simulation in Intergranular Corrosion

**Figure 36:**
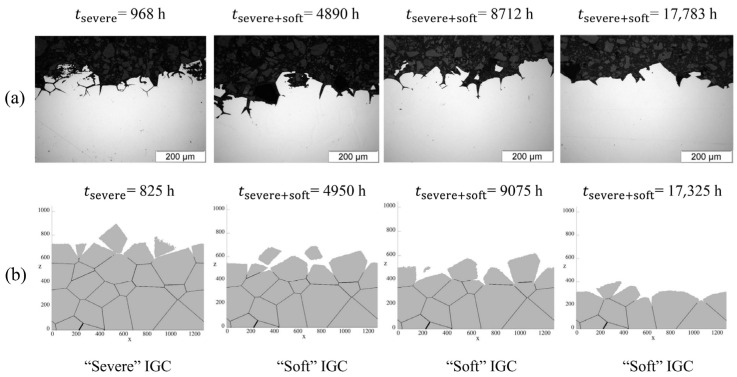
Evolution of the IGC on cross-sections in the case of alternation a “severe” and a “soft” IGC: (**a**) experiment; (**b**) CA simulations [110].

Combining the different corrosion kinetics, Guiso et al. [108,109] proposed a three-dimensional CA model to investigate the intergranular corrosion (IGC) phenomenon. Moreover, the CA model provided realistic morphological features of IGC, such as groove-shaped corrosion and grain detachment [108]. In order to investigate the impact of the oxidizing character of the nitric medium on the evolution of the intergranular corrosion of 310L stainless steel, Guiso et al. [110] also used the results of the CA simulations to quantify precisely the effect of the oxidizing medium on the IGC of the stainless steel. Figure 36 shows the evolution of the IGC on cross-sections in the case of alternation between a “severe” and a “soft” IGC. The system re-adapted to the “soft” conditions without memory effect from the previous “severe” ones [110].

In brief, various CA models of metal corrosion can predict the evolution of corrosion rate, morphology, etc., which provide effective modeling support for the development of better corrosion-protection strategies.

## 4. Future Research Directions of CA in Metallic Materials Research

Cellular automata, as a tool for multi-scale simulation of the microstructure evolution of metallic materials, has gradually matured in the applications of solidification, recrystallization and phase transformation research. Recently, the CA method has also developed rapidly in the research directions of additive manufacturing, corrosion and protection, etc. In fact, the development and application of CA simulation are full of challenges, and there are still many problems that need to be further studied and developed. Using CA to assist in simulating the crystal structure and predicting the mechanical properties of metallic materials must be addressed urgently. In addition, in the research of metallic materials, cellular automata themselves also needs to move forward. It is expected that the main directions are development of CA simulation general software, rule systems, etc.

### 4.1. Auxiliary Simulation of Crystal Structure of Metallic Materials by CA

A smaller level than the grain size is the crystal structure of the material. Crystal structure involves lattice, orientation, texture, slip, etc. These concepts have been deeply studied, and a great deal of data have been accumulated in crystal plasticity mechanics. Various mathematical models have been established to reveal the micro-nature of metallic materials and the deep law of plastic deformation. CA are also needed here to provide relevant graphics and image information. These graphics and image information can provide the traditional mathematical models with the exact image and assist people to recognize the essence through phenomena and discover the rules.

The combination of CA and crystal plasticity is a good research direction. The simulation results of crystal plasticity show that the crystal plastic deformation is extremely inhomogeneous, and there are some zones where the orientation changes dramatically [111,112]. According to CA theory, this is the most likely nucleation area, which is consistent with the experimental observations. The combination of CA and crystal plasticity can explain other similar experimental phenomena. The research work in this area has broad prospects for development.

### 4.2. Prediction of Mechanical Properties of Metallic Materials by CA

The mechanical properties of metallic materials are determined by their chemical composition, processing and organizational microstructure. Under the determined chemical composition and processing conditions, the mechanical properties are determined by the microstructure. The current study has shown that CA is able to simulate the change in dislocation density in dynamic recrystallization. Since there is a relationship between flow stress and dislocation density, the flow stress during thermal deformation is also obtained via CA simulation [65,113,114,115]. Since CA can reasonably simulate the microstructure of metallic materials, it is no longer far from predicting the mechanical properties of metallic materials.

The famous Hall–Petch equation helps us to establish the relationship between the microstructure and properties of metallic materials [116,117,118]. A simple method is to obtain the information of grain size by using CA, which is brought into the Hall–Petch equation to calculate mechanical properties such as yield strength. Here, grain size is an average, equivalent and approximate concept. If we make a further study and take fully into account more abundant information about grain shape, grain size, grain axis ratio, grain size distribution, grain boundary and grain orientation provided by CA, we will establish a set of more precise mathematical models to describe the relationship between mechanical properties, and structure can be established. The realization of cellular automata in the prediction of mechanical properties may be the integration of machine-learning algorithms and data-driven approaches [119,120]. This integration leads to more accurate predictive models by large datasets and optimizing model parameters based on experimental data [121,122]. This research will open a new chapter in the study of mechanical properties and raise our understanding of the nature of mechanical properties of metallic materials to a new level.

### 4.3. Development and Optimization of CA Simulation Software

(1) Development of CA general software

At present, the CA method is used to simulate microstructure evolution processes and visual results by writing the simulation program using MATLAB, PYTHON, FORTRAN or other program languages. There is no complete and systematic CA commercial software that can be used to simulate microstructure evolution during the forming process of metallic materials, and the finite element method has yielded a successful example for us. The finite element commercial software for metal forming analysis includes ANSYS, MARC, DEFORM, ABAQUS, etc., which forms a software platform system for users to choose according to their solution needs. Developing a series of named commercial software is a powerful measure to speed up the popularization and application of CA. If scholars can develop commercial CA simulation software like ANSYS, which is convenient for beginners to use, CA would be more widely and deeply applied to the simulation research of different aspects in the field of metallic materials.

Standardization of program, modularization of software and practicality of function are the only way to develop commercial software, and the development of CA commercial software is no exception. Through the efforts of researchers, we hope that we can follow the unified standard and develop all kinds of CA source program modules with clear division of labor, complete functions and a self-contained system. We look forward to buying the ideal commercial software for cellular automata in the market in the near future. Once the beginners have mastered the foundation knowledge of CA, they will no longer be needed to spend time on programming and debugging and will concentrate more energy on the creative work of CA application.

(2) Development of the CA rule system and the new CA model

CA works based on rules. It can be said that rules play a very important role in CA. Existing rules play a key role in the application of various CA and have been verified in the simulation of metal solidification, recrystallization and phase transformation.

Developing new rules without sticking to the existing rules system is a way for CA to deal with new problems and put forward new ideas for further development. The fields of metallic materials science shows a variety of rich phenomena and contains different deep laws. It needs to be revealed from different perspectives and described by different rules. For example, the question remains whether different rules can be established to describe edge dislocation and screw dislocation reasonably, simulate the climbing, pinning, increment and disappearance of dislocation, provide visual graphics and image information of dislocation movement and deepen our understanding and understanding of dislocation movement and even plastic deformation.

Based on the existing rule system, the existing models of CA are developed. The establishment of a new rule system will inevitably lead to the emergence of new models of CA, which are powerful tools for us to deal with new scientific problems. We eagerly hope that this day will come soon.

(3) Development of pre- and post-processing functions of CA

A good commercial software should be equipped with a user-friendly man–machine interface, which cannot be separated from good pre- and post-processing functions. CA have prominent features of graphics and images, and the human–machine interface is more important. The simulation results of CA are given in two ways: graph and data. The final morphology of solidification, recrystallization and phase transformation products should be given as well as the complete data describing their characteristics.

For example, after completing CA simulation of the recrystallization process, not only can we obtain a graph of grain distribution and additional information such as orientation by color, but also the size distribution of each grain, the axis ratio of grain shape, the polar gram reflecting grain orientation and the average grain size; minimum and maximum grain characteristic parameter and orientation difference can also be obtained.

In addition to the final results, the study of CA is concerned with the process of obtaining these final results. Many parameters in metallic materials are related not only to time, but also to process. In other words, state functions are process variables, and CA often leave traces of processes when describing states. The whole process recorded by CA is displayed truthfully, continuously and dynamically, showing us not only a fixed figure and picture, but also a continuous image changing over time. These images are precious to researchers. Perhaps we can discover some clues from development and innovation to help us understand the essence of things. Just like on a journey, using a camera to record the scenery along the way will leave us a deeper, more vivid and more unforgettable impression.

It can be seen that recording and reproducing the process is very important for CA simulation. In the future, the human–machine interface of CA should not only give the final results, but also give the complete process of obtaining these results. In the scientific research of metallic materials, we should give enough attention to the interpretation process of CA.

## 5. Summary

Cellular automata have been widely used in the microstructure simulation of metallics research, which has been an effective method to explore the law and mechanism governing microstructure evolution. In this paper, we introduce the advantages and limitations of CA compared to other widely used simulation programs. Subsequently, we present an overview of the research progress of CA in the microstructure simulation of metal solidification, recrystallization, phase transformation and carbide precipitation during forming and heat treatment in the last five years. At the same time, we also elucidate the recent research advances in microstructure simulation via CA in the fields of additive manufacturing, welding, asymmetrical rolling, corrosion prevention, etc. Finally, we point out potential future research directions for CA in the field of materials science, especially in the simulation of the crystal structure, the prediction of mechanical properties, CA simulation software and rule systems, etc. The application of CA in metallic materials science shows researchers a new and colorful sky. What we need to continue to explore is far more profound, more mysterious and more magical than what we have at hand. The research work of this paper will promote the development of CA in materials research, which is expected to attract widespread attention of researchers in the field of research and development of metallic materials.

## References

[1] Su Y.J., Fu H.D., Bai Y., Jiang X., Xie J.X. (2020). Progress in materials genome engineering in China. Acta Metall. Sin..

[2] Wang H., Xiang Y., Xiang X.D., Chen L.Q. (2015). Materials genome enables research and development revolution. Sci. Technol. Rev..

[3] Yang H., Wu C., Li H.W., Fan X.G. (2011). Review on cellular automata simulations of microstructure evolution during metal forming process: Grain coarsening, recrystallization and phase transformation. Sci. China Technol. Sci..

[4] Chen F., Zhu H.J., Chen W., Ou H.G., Cui Z.S. (2019). Multiscale modeling of discontinuous dynamic recrystallization during hot working by coupling multilevel cellular automaton and finite element method. Int. J. Plast..

[5] Rezaei J., Parsa M.H., Mirzadeh H. (2023). Phase transformation kinetics of high-carbon steel during continuous heating. J. Mater. Res. Technol..

[6] Dorari E., Ji K., Guillemot G., Gandin C.A., Karma A. (2022). Growth competition between columnar dendritic grains—The role of microstructural length scales. Acta Mater..

[7] Su F., Liu W.L., Wen Z. (2020). Three-dimensional cellular automaton simulation of austenite grain growth of Fe-1C-1.5Cr alloy steel. J. Mater. Res. Technol..

[8] Zenkri M., di Caprio D., Raouafi F., Féron D. (2022). Cathodic control using cellular automata approach. Mater. Corros..

[9] Jin S.D., He J.P., Pan X.H. (2021). Progress in research methods of microstructure evolution during welding solidification. J. Shanghai Univ. Eng. Sci..

[10] Xin Q.B. (2013). Computer Simulation of Material Forming.

[11] Yin H.B., Felicelli S.D. (2009). A cellular automaton model for dendrite growth in magnesium alloy AZ91. Model. Simul. Mater. Sci. Eng..

[12] Miodownik M.A. (2002). A review of microstructural computer models used to simulate grain growth and recrystallisation in aluminium alloys. J. Light Met..

[13] Wang W., Helbert A.L., Brisset F., Mathon M.H., Baudin T. (2014). Monte Carlo simulation of primary recrystallization and annealing twinning. Acta Mater..

[14] Radhakrishnan B., Sarma G., Zacharia T. (1998). Modeling of Nucleation during Recrystallization.

[15] Wang K.Y., Lv S.J., Wu H.H., Wu G.L., Wang S.Z., Gao J.H., Zhu J.M., Yang X.S., Mao X.P. (2023). Recent research progress on the phase-field model of microstructural evolution during metal solidification. Int. J. Miner. Metall. Mater..

[16] Ren X., Dou C.Y., Gao Z.Y., Zhuang D., Qi P.T., He W. (2021). Research progress of numerical simulation in heat treatment. Mater. Rep..

[17] Raabe D. (2003). Computational Materials Science.

[18] Körner C., Markl M., Koepf J.A. (2020). Modeling and simulation of microstructure evolution for additive manufacturing of metals: A critical review. Metall. Mater. Trans. A.

[19] Grilli N., Janssens K.G.F., Swygenhoven H.V. (2015). Crystal plasticity finite element modelling of low cycle fatigue in FCC metals. J. Mech. Phys. Solids.

[20] Kohar C.P., Bassani J.L., Brahme A., Muhammad W., Mishra R.K., Inal K. (2019). A new multi-scale framework to incorporate microstructure evolution in phenomenological plasticity: Theory, explicit finite element formulation, implementation and validation. Int. J. Plast..

[21] Casals O., Ocenasek J., Alcala J. (2007). Crystal plasticity finite element simulations of pyramidal indentation in copper single crystals. Acta Mater..

[22] Zhou X.R., He L., Zhou T., Jiang H.W., Xu J.Y., Tian P.F., Zou Z.C., Du F.L. (2022). Multiscale research of microstructure evolution during turning Ti-6Al-4V alloy based on FE and CA. J. Alloys Compd..

[23] Koepf J.A., Soldner D., Ramsperger M., Mergheim J., Markl M., Körner C. (2019). Numerical microstructure prediction by a coupled finite element cellular automaton model for selective electron beam melting. Comput. Mater. Sci..

[24] Li B.K., Wang Q., Wang F., Chen M.Q. (2014). A coupled cellular automaton–finite-element mathematical model for the multiscale phenomena of electroslag remelting H13 die steel ingot. JOM.

[25] Guo X., Sun Q.Q., Yang T., Weng G.J., Zhang C.B., Feng X.Q. (2018). Local Monte Carlo method for fatigue analysis of coarse-grained metals with a nanograined surface layer. Metals.

[26] Cao L., Zhang L., Meng R.F., Zhang Q.D. (2022). Analyzing effects of temperature gradient and scan rate on metal additive manufacturing microstructure by using phase field-finite element method. Model. Simul. Mater. Sci. Eng..

[27] Zhang Z., Wang Y.F., Ge P., Wu T. (2022). A review on modelling and simulation of laser additive manufacturing: Heat transfer, microstructure evolutions and mechanical properties. Coatings.

[28] Zhang Z., Tan Z.J., Yao X.X., Hu C.P., Ge P., Wan Z.Y., Li J.Y., Wu Q. (2019). Numerical methods for microstructural evolutions in laser additive manufacturing. Comput. Math. Appl..

[29] Sieradzki L., Madej L. (2013). A perceptive comparison of the cellular automata and Monte Carlo techniques in application to static recrystallization modeling in polycrystalline materials. Comput. Mater. Sci..

[30] Zhu Y.C., Zhong S.J., Guo J.H., Qin J., Xu H., Yang Y. (2023). Research progress on phase field simulation of microstructure evolution in welding process. Electr. Weld. Mach..

[31] Wang X.Y., Lu Q.H., Zhang P.L., Yan H., Shi H.C., Sun T.Z., Zhou K., Chen K.Y. (2024). A review on the simulation of selective laser melting AlSi10Mg. Opt. Laser Technol..

[32] Bailey N.S., Shin Y.C. (2022). Multi-track, multi-layer dendrite growth and solid phase transformation analysis during additive manufacturing of H13 tool steel using a combined hybrid cellular automata/phase field, solid-state phase prediction models. Int. J. Adv. Manuf. Technol..

[33] Tang J., Kumar S., Lorenzis L.D., Hosseini E. (2023). Neural cellular automata for solidification microstructure modelling. Comput. Methods Appl. Mech. Eng..

[34] Pineau A., Guillemot G., Tourret D., Karma A., Gandin C.A. (2018). Growth competition between columnar dendritic grains—Cellular automaton versus phase field modeling. Acta Mater..

[35] Tourret D., Song Y., Clarke A.J., Karma A. (2017). Grain growth competition during thin-sample directional solidification of dendritic microstructures: A phase-field study. Acta Mater..

[36] Tourret D., Karma A. (2015). Growth competition of columnar dendritic grains: A phase-field study. Acta Mater..

[37] Chen S.J., Guillemot G., Gandin C.A. (2016). Three-dimensional cellular automaton-finite element modeling of solidification grain structures for arc-welding processes. Acta Mater..

[38] Sitko M., Madej L. (2021). The role of the cellular automata cell size and time step length in the microstructure evolution model—The static recrystallization case study. J. Comput. Sci..

[39] Zhu H.J., Chen F., Zhang H.M., Cui Z.S. (2020). Review on modeling and simulation of microstructure evolution during dynamic recrystallization using cellular automaton method. Sci. China Technol. Sci..

[40] Packard N. (1986). Lattice Models for Solidification and Aggregation.

[41] Bai C.F., Wang B., Ma J., Zhang J.Y., Pan W.P. (2023). Modeling effect of cooling conditions on solidification process during thermal cycle of rollers in twin-roll strip casting. J. Iron Steel Res. Int..

[42] Wang W.L., Liu Y.H., Guo L.T., Wang Y.L. (2022). Cellular automaton simulation of dendrite growth in solidification process of Cr17 stainless steel under mechanical vibration. Phys. Status Solidi A.

[43] Ridgeway C.D., Gu C., Ripplinger K., Detwiler D., Ji M.S., Sohgrati S., Luo A.A. (2020). Prediction of location specific mechanical properties of aluminum casting using a new CA-FEA (cellular automaton-finite element analysis) approach. Mater. Des..

[44] Hu M.D., Wang T.T., Fang H., Zhu M.F. (2021). Modeling of gas porosity and microstructure formation during dendritic and eutectic solidification of ternary Al-Si-Mg alloys. J. Mater. Sci. Technol..

[45] Qin Q., Ye C.L., Xie L., Wang T. (2022). Dendrite growth state under non-uniform temperature field. Rare Met. Mater. Eng..

[46] Wang X.Y., Wang F.F., Wu K.Y., Wang X.F., Xiao L., Li Z.Q., Han Z.Q. (2021). Experimental study and cellular automaton simulation on solidification microstructure of Mg-Gd-Y-Zr alloy. Rare Met..

[47] Liang X.H., Bos C., Hermans M., Richardson I. (2023). An improved cellular automata solidification model considering kinetic undercooling. Metall. Mater. Trans. B.

[48] Lee W., Bae J., Lee H., Kang S.H., Yoon J. (2022). Numerical simulation of dendritic growth and porosity evolution in solidification of Al-Cu alloy with lattice Boltzmann-Cellular automata method. J. Alloys Compd..

[49] Gu C., Moodispaw M.P., Luo A.A. (2022). Cellular automaton simulation and experimental validation of eutectic transformation during solidification of Al-Si alloys. npj Comput. Mater..

[50] Gu H., Väistö T., Wei C., Li L., Ren X.D., Qian L.L. (2023). A coupled ray-tracing based CFD and cellular automaton model for predicting molten pool formation and microstructure evolution in narrow gap laser welding. Int. J. Heat Mass Transf..

[51] Yang Z.Y., Fang H., Jin K.N., He J.S., Ge W.J., Yan W.T. (2022). Physics-driven modeling of electron beam welding of Al-Cu alloys from molten pool flow, microstructure to mechanical properties. J. Mater. Process. Technol..

[52] Chen Y., Chen X.M., Zhang Q.Y., Zhu M.F., Liu F., Wang X.N., Nagaumi H., Yao Z.J. (2022). Numerical modeling of welding porosity formation and dendrite growth of 6xxx aluminum alloys. JOM.

[53] Feng H.J., Yan X., Tang Y.F., Dong Y. (2014). Summary of 3D printing technology. Digit. Technol. Appl..

[54] Wang Y., Liu Y.M., Liu J.W., Wei Y.K., Zhang L.L., Wang J.Y., Shang W.W., Liu S.F. (2022). Research progress on numerical simulation of metal additive-manufacturing process. Powder Metall. Technol..

[55] Yu Y.F., Li Y., Lin F., Yan W.T. (2021). A multi-grid cellular automaton model for simulating dendrite growth and its application in additive manufacturing. Addit. Manuf..

[56] Meng G.R., Gong Y.D., Zhang J.D., Zhu L.D., Xie H.L., Zhao J.B. (2022). Multi-scale simulation of microstructure evolution during direct laser deposition of Inconel 718. Int. J. Heat Mass Transf..

[57] Zhang Y., Zhang J. (2019). Modeling of solidification microstructure evolution in laser powder bed fusion fabricated 316L stainless steel using combined computational fluid dynamics and cellular automata. Addit. Manuf..

[58] Yadroitsev I., Krakhmalev P., Yadroitsava I., Johansson S., Smurov I. (2013). Energy input effect on morphology and microstructure of selective laser melting single track from metallic powder. J. Mater. Process. Technol..

[59] Gandin C.A., Rappaz M. (1997). A 3D Cellular Automaton algorithm for the prediction of dendritic grain growth. Acta Mater..

[60] Hesselbarth H.W., Gobel I.R. (1991). Simulation of recrystallization by cellular automata. Acta Metall. Mater..

[61] Goetz R.L., Seetharaman V. (1998). Static recrystallization kinetics with homogeneous heterogeneous nucleation using a cellular automata model. Metall. Mater. Trans. A.

[62] Goetz R.L., Seetharaman V. (1998). Modeling dynamic recrystallization using cellular automata. Scr. Mater..

[63] Li F., Zhang L.W., Zhang C., Song K.J., Mao P.G. (2022). Numerical simulation on recrystallization behavior and microstructure evolution during hot continuous rolling process of 38CrMoAl steel rod. J. Iron Steel Res. Int..

[64] Shah V., Sedighiani K., Van Dokkum J.S., Bos C., Roters F., Diehl M. (2022). Coupling crystal plasticity and cellular automaton models to study meta-dynamic recrystallization during hot rolling at high strain rates. Mater. Sci. Eng..

[65] Alone A., Chatterjee R., Alankar A. (2020). A comparative study of the effect of random and preferred crystallographic orientations on dynamic recrystallization behavior using a cellular automata model. Mater. Today Commun..

[66] Lin Y.C., Wen D.X., Chen M.S., Chen X.M. (2016). A novel unified dislocation density-based model for hot deformation behavior of a nickel-based superalloy under dynamic recrystallization conditions. Appl. Phys. A.

[67] Guan X.J., Yu B.J. (2017). Cellular automaton simulation for the effects of uneven distribution of dislocation density and small-sized precipitated particles on dynamic recrystallization. IOP Conf. Ser. Mater. Sci. Eng..

[68] Wang Y., Xing X.D., Zhang Y.Q., Jiang S.Y. (2019). Investigation of the dynamic recrystallization of FeMnSiCrNi shape memory alloy under hot compression based on cellular automaton. Metals.

[69] Cao Z.H., Sun Y., Zhou C., Wan Z.P., Yang W.H., Ren L.L., Hu L.X. (2019). Cellular automaton simulation of dynamic recrystallization behavior in V-10Cr-5Ti alloy under hot deformation conditions. Trans. Nonferr. Met. Soc. China.

[70] Lu R.Q., Zheng S.W., Teng J., Hu J.M., Fu D.F., Chen J.C., Zhao G.D., Jiang F.L., Zhang H. (2021). Microstructure, mechanical properties and deformation characteristics of Al-Mg-Si alloys processed by a continuous expansion extrusion approach. J. Mater. Sci. Technol..

[71] Yao Y.L., Xiu S.C., Sun C., Kong X.N., Hong Y. (2021). Investigation on grinding-induced dynamic recrystallization behavior of 40Cr alloy steel. J. Alloys Compd..

[72] Duan X.W., Wang M., Che X., He L.F., Liu J.C. (2023). Cellular automata coupled finite element simulation for dynamic recrystallization of extruded AZ80A magnesium alloy. J. Mater. Sci..

[73] Hong Y., Sun C., Xiu S.C., Yao Y.L., Chen X. (2021). Investigation on dynamic recrystallization behavior of abrasive grinding hardening surface. Surf. Technol..

[74] Yang Y.Q., Zhang W.W., Tan Y.B., Yan K.Q., Shu J., Luo T., Lu C.H. (2021). Simulation of dynamically recrystallized structure of TB8 titanium alloy during hot reduction. Heat Treat..

[75] Kushwaha R., Muhammad W., Ali U., Sahoo S.K., Sabat R.K. (2023). Effect of solute concentration on microstructure evolution during static recrystallization in Mg-0.2%Ce alloy using cellular automata. Mater. Today Commun..

[76] Asgharzadeh A., Tiji S.A.N., Park T., Kim J.H., Pourboghra F. (2020). Cellular automata modeling of the kinetics of static recrystallization during the post-hydroforming annealing of steel tube. J. Mater. Sci..

[77] Asgharzadeh A., Tiji S.A.N., Park T., Pourboghra F. (2022). Prediction of softening kinetics and recrystallization texture in non-isothermally annealed bulged tubes using CPFEM and CA models. Mater. Sci. Eng. A.

[78] Zhang T., Li L., Lu S.H., Zhang J.B., Gong H. (2018). Comparisons of flow behavior characteristics and microstructure between asymmetrical shear rolling and symmetrical rolling by macro/micro coupling simulation. J. Comput. Sci..

[79] Sun H., Chen M., Cheng M., Wang R.X., Wang X., Hu X.D., Zhao H.Y., Ju D.Y. (2021). A multi-scale model for elucidation of recrystallization and texture of Mg-Alloy sheet by warm-rolling process. Chin. J. Mater. Res..

[80] Zhang T., Li L., Lu S.H., Gong H., Wu Y.X. (2018). Comparisons of different models on dynamic recrystallization of plate during asymmetrical shear rolling. Materials.

[81] Shen Y., Gu Z.M., Wang C. (2023). Phase transformation behaviors in the heat-affected zones of ferritic heat-resistant steels enabled by in situ CSLM observation. Acta Metall. Sin..

[82] Kumar M., Sasikumar R., Kesavan Nair P. (1998). Competition between nucleation and early growth of ferrite from austenite-studies using cellular automata. Acta Mater..

[83] Lin X., Zou X.Y., An D., Krakauer B.W., Zhu M.F. (2021). Multi-scale modeling of microstructure evolution during multi-pass hot-rolling and cooling process. Materials.

[84] Li X.H., Zhang Y., Liu Y.C., Gan K.F., Liu C.X. (2020). Multi-phase transformation kinetics of HSLA steels during continuous cooling: Experiments and cellular automaton (CA) simulation. Philos. Mag..

[85] Łach Ł., Svyetlichnyy D. (2020). Development of hybrid model for modeling of diffusion phase transformation. Eng. Comput..

[86] Łach Ł., Svyetlichnyy D. (2023). 3D model of heat flow during diffusional phase transformations. Materials.

[87] Duan C.Z., Kou W.N., Zhang F.Y. (2019). Cellular automata simulation for phase transition of surface white layer in high-speed dry cutting. Tool Eng..

[88] Seppälä O., Pohjonen A., Kaijalainen A., Larkiola J., Porter D. (2018). Simulation of bainite and martensite formation using a novel cellular automata method. Procedia Manuf..

[89] Vijay Reddy K., Halder C., Pal S. (2017). Influence of carbon equivalent content on phase transformation during inter-critical heating of dual phase steels using discrete micro-scale cellular automata model. Trans. Indian Inst. Met..

[90] Halder C., Madej L., Pietrzyk M. (2014). Discrete micro-scale cellular automata model for modelling phase transformation during heating of dual phase steels. Arch. Civ. Mech. Eng..

[91] Jia C.N., Zheng C.W., Li D.Z. (2020). Cellular automaton modeling of austenite formation from ferrite plus pearlite microstructures during intercritical annealing of a C-Mn steel. J. Mater. Sci. Technol..

[92] Yu Y.F., Kenevisi M.S., Yan W.T., Lin F. (2020). Modeling precipitation process of Al-Cu alloy in electron beam selective melting with a 3D cellular automaton model. Addit. Manuf..

[93] Yang J.J., Yu H.C., Yang H.H., Li F.Z., Wang Z.M., Zeng X.Y. (2018). Prediction of microstructure in selective laser melted Ti-6Al-4V alloy by cellular automaton. J. Alloys Compd..

[94] Marynowski P., Adrian H., Głowacki M. (2019). Modeling of the kinetics of carbonitride precipitation process in high-strength low-alloy steels using cellular automata method. J. Mater. Eng. Perform..

[95] Svyetlichnyy D.S. (2020). Development of precipitation model with the use of the Lattice Boltzmann Method and its application for the rolling process. Comput. Methods Appl. Mech. Eng..

[96] Przemyslaw M., Henryk A., Miroslaw G., Krzysztof W. Cellular automata model of carbonitride precipitation process to simulate image of microstructure in microalloyed steels. Proceedings of the METAL 2017: 26th International Conference on Metallurgy and Materials.

[97] Zhang S., Zhang H.W., Miao M., Feng M.M., Lei H., Wang Q. (2021). Cellular automaton simulation on cooperative growth of M_7_C_3_ carbide and austenite in high Cr cast irons. Acta Phys. Sin..

[98] Zhang H.W., Zhang S., Wang Y.C., Hao Y.Z., Miao M., Nakajima K., Lei H., Wang Q., He J.C. (2020). Cellular automaton modelling of M_7_C_3_ carbide growth during solidification of Fe-C-Cr alloy. IOP Conf. Ser. Mater. Sci. Eng..

[99] Wang W.L., Guan B., Wei X.L., Lu J.F., Ding J. (2019). Cellular automata simulation on the corrosion behavior of Ni-base alloy in chloride molten salt. Sol. Energy Mater. Sol. Cells.

[100] Guo Y., Tian G., Liu D.J., Zhang Y.H., Chang X.L. (2022). Corrosion behavior of aluminum lithium alloys in acidic environment and cellular automata simulation. China Mech. Eng..

[101] Chen Z., Sun L., Zhang W., Zheng H., Xia W., Zeng H., Chen S., Li K., Li W. (2022). Corrosion behavior of marine structural steel in tidal zone based on wire beam electrode technology and partitioned cellular automata model. Corros. Commun..

[102] Xu Z.H., Lu J.F., Wei X.L., Ding J., Wang W.L. (2022). 2D and 3D cellular automata simulation on the corrosion behaviour of Ni-based alloy in ternary molten salt of NaCl–KCl–ZnCl_2_. Sol. Energy Mater. Sol. Cells.

[103] Liu X.F., Hua L., Chang D.M. (2021). Simulation of pit interactions of multi-pit corrosion under an anticorrosive coating with a three-dimensional cellular automata model. Model. Simul. Mater. Sci. Eng..

[104] Wang H., Han E.H. (2016). Computational simulation of corrosion pit interactions under mechanochemical effects using a cellular automaton/finite element model. Corros. Sci..

[105] Rusyn B.P., Tors’Ka R.V., Pokhmurs’Kyi A.Y. (2015). Modeling of the evolution of corrosion pitting with the use of cellular automata. Mater. Sci..

[106] Zhang X., Hu J., Wang Y., Zheng M., Zhang Z. (2015). Simulation of pitting corrosion for Ni-based alloy using a cellular automata model. Rare Met. Mater. Eng..

[107] Cui C.J., Ma R.J., Chen A.R., Pan Z.C., Tian H. (2019). Experimental study and 3D cellular automata simulation of corrosion pits on Q345 steel surface under salt-spray environment. Corros. Sci..

[108] Guiso S., Caprio D.D., Lamare J.D., Gwinner B. (2020). Intergranular corrosion: Comparison between experiments and cellular automata. Corros. Sci..

[109] Guiso S., di Caprio D., de Lamare J., Gwinner B. (2021). Influence of the grid cell geometry on 3D cellular automata behavior in intergranular corrosion. J. Comput. Sci..

[110] Guiso S., Brijou Mokrani N., de Lamare J., Di Caprio D., Gwinner B., Lorentz V., Miserque F. (2022). Intergranular corrosion in evolving media: Experiment and modeling by cellular automata. Corros. Sci..

[111] Chen S.D., Liu X.H., Liu L.Z. (2015). Symmetric and asymmetric rolling pure copper foil: Crystal plasticity finite element simulation and experiments. Acta Metall. Sin..

[112] Cheng J.H., Lin B.K., Pottore N.S., Sadagopan S., Zhu H., Hu X.H. (2024). A mesoscale crystal plasticity model to predict room-temperature deformation and martensitic transformation of high-strength quenching and partitioning (QP) Steels and validation with synchrotron X-ray diffraction. Int. J. Plast..

[113] Liu L., Wu Y.X., Ahmad A.S. (2021). A novel simulation of continuous dynamic recrystallization process for 2219 aluminium alloy using cellular automata technique. Mater. Sci. Eng. A.

[114] Mei M.L., Song Y.L., Lu J., Hao C.C., Xie L.C. (2022). Modeling dynamic recrystallization behavior of Al-Zn-Mg-Cu alloy during electroshock assisted tension based on cellular automata. Mater. Res. Express.

[115] Zhi Y., Liu X.H., Yu H.L. (2014). Cellular automaton simulation of hot deformation of TRIP steel. Comput. Mater. Sci..

[116] Ren R., Fan J.F., Wang B.S., Zhang Q., Li W.G., Dong H.B. (2022). Hall-Petch relationship and deformation mechanism of pure Mg at room temperature. J. Alloys Compd..

[117] Liu L., Zhou X.J., Yu S.L., Zhang J., Lu X.Z., Shu X., Su Z.J. (2022). Effects of heat treatment on mechanical properties of an extruded Mg-4.3Gd-3.2Y-1.2Zn-0.5Zr alloy and establishment of its Hall–Petch relation. J. Magnes. Alloys.

[118] Kwak T.Y., Kim W.J. (2019). Mechanical properties and Hall-Petch relationship of the extruded Mg-Zn-Y alloys with different volume fractions of icosahedral phase. J. Alloys Compd..

[119] Shen C.G., Wang C.C., Wei X.L., Li Y., van der Zwaag S., Xu W. (2019). Physical metallurgy-guided machine learning and artificial intelligent design of ultrahigh-strength stainless steel. Acta Mater..

[120] Wang C.C., Wei X.L., Ren D., Wang X., Xu W. (2022). High-throughput map design of creep life in low-alloy steels by integrating machine learning with a genetic algorithm. Mater. Des..

[121] Hu M.W., Tan Q.Y., Knibbe R., Xu M., Jiang B., Wang S., Li X., Zhang M.X. (2023). Recent applications of machine learning in alloy design: A review. Mater. Sci. Eng. R Rep..

[122] Ren D., Wang C.C., Wei X.L., Lai Q.Q., Xu W. (2023). Building a quantitative composition-microstructure-property relationship of dual-phase steels via multimodal data mining. Acta Mater..

